# Unlocking potential biomarkers bridging coronary atherosclerosis and pyrimidine metabolism-associated genes through an integrated bioinformatics and machine learning approach

**DOI:** 10.1186/s12872-024-03819-w

**Published:** 2024-03-07

**Authors:** Fanli Bu, Xiao Qin, Tiantian Wang, Na Li, Man Zheng, Zixuan Wu, Kai Ma

**Affiliations:** 1 Dongying People’s Hospital (Dongying Hospital of Shandong Provincial Hospital Group), Dongying, Shandong 257091 People’s Republic of China; 2 Guangzhou University of Chinese Medicine, Guangzhou, China

**Keywords:** Atherosclerosis (AS), Pyrimidine Metabolism Genes (PyMGs), Lasso regression, SVM-RFE, Bioinformatics

## Abstract

**Background:**

This study delves into the intricate landscape of atherosclerosis (AS), a chronic inflammatory disorder with significant implications for cardiovascular health. AS poses a considerable burden on global healthcare systems, elevating both mortality and morbidity rates. The pathological underpinnings of AS involve a marked metabolic disequilibrium, particularly within pyrimidine metabolism (PyM), a crucial enzymatic network central to nucleotide synthesis and degradation. While the therapeutic relevance of pyrimidine metabolism in diverse diseases is acknowledged, the explicit role of pyrimidine metabolism genes (PyMGs) in the context of AS remains elusive. Utilizing bioinformatics methodologies, this investigation aims to reveal and substantiate PyMGs intricately linked with AS.

**Methods:**

A set of 41 candidate PyMGs was scrutinized through differential expression analysis. GSEA and GSVA were employed to illuminate potential biological pathways and functions associated with the identified PyMGs. Simultaneously, Lasso regression and SVM-RFE were utilized to distill core genes and assess the diagnostic potential of four quintessential PyMGs (CMPK1, CMPK2, NT5C2, RRM1) in discriminating AS. The relationship between key PyMGs and clinical presentations was also explored. Validation of the expression levels of the four PyMGs was performed using the GSE43292 and GSE9820 datasets.

**Results:**

This investigation identified four PyMGs, with NT5C2 and RRM1 emerging as key players, intricately linked to AS pathogenesis. Functional analysis underscored their critical involvement in metabolic processes, including pyrimidine-containing compound metabolism and nucleotide biosynthesis. Diagnostic evaluation of these PyMGs in distinguishing AS showcased promising results.

**Conclusion:**

In conclusion, this exploration has illuminated a constellation of four PyMGs with a potential nexus to AS pathogenesis. These findings unveil emerging biomarkers, paving the way for novel approaches to disease monitoring and progression, and providing new avenues for therapeutic intervention in the realm of atherosclerosis.

**Supplementary Information:**

The online version contains supplementary material available at 10.1186/s12872-024-03819-w.

## Introduction

Coronary artery disease (CAD), a leading cause of global mortality, is projected to cause 23.4 million deaths by 2030, up from 18 million in 2017 [1]. This alarming trend is partly attributed to diets high in fats and sugars but low in fiber, exacerbating AS prevalence [2]. AS, a chronic inflammatory arterial disease, commences with inflammation in the arterial intima and is characterized by the formation of atherosclerotic plaques, driven by LDL invasion and retention, and the aggregation of inflammatory cells [2]. This process underpins the pathogenesis of CAD, leading to coronary obstruction, myocardial ischemia, and necrosis, thereby heightening the risk of ACS. Despite extensive research, the genomic regulatory mechanisms of AS remain elusive [3]. Metabolism, a complex network of biochemical reactions, is increasingly recognized as a key player in the pathogenesis and progression of various diseases, including metabolic, oncological, and cardiovascular disorders [4]. Disrupted metabolic pathways are a hallmark of disease pathology. Advances in metabolic research have shed light on the interplay between metabolic processes and disease development, identifying potential therapeutic targets and biomarkers [5]. Moreover, metabolism is critical in modulating immune responses and inflammatory conditions. It shapes immune cell function, influencing their differentiation, activation, and efficacy [6]. Alterations in immune cell metabolism can lead to autoimmune disorders, sustained inflammatory states, and weakened defenses against pathogens [7]. This study endeavors to demystify the complex relationship between coronary artery disease, atherosclerosis, and metabolic dysfunction. By exploring the genomic regulatory complexities of atherosclerosis and the impact of metabolic pathways on disease progression, this research aims to open new avenues for therapeutic interventions and precision medicine in cardiovascular health [7].

Nutrient assimilation and metabolic flux are essential for the survival of all living organisms. In oncology, metabolic reprogramming is a critical factor, influencing tumor proliferation and survival. Recent research has revealed that oncogenic transformation imparts a unique metabolic signature to tumor cells, altering the tumor microenvironment (TME). The TME, comprising various cell types within a complex extracellular matrix, often suffers from poor oxygen and nutrient distribution due to underdeveloped or abnormal vasculature. As research advances, examining immune infiltration in non-tumorigenic areas is becoming increasingly vital [8]. There is growing evidence that the immune response is closely connected to significant metabolic changes in tissues, leading to nutrient depletion, increased oxygen consumption, and the production of reactive nitrogen and oxygen species [8]. Additionally, various factors within the TME significantly affect the proliferation and function of immune cells. This interplay suggests that metabolic interventions could enhance the effectiveness of immunotherapeutic approaches in cancer. Consequently, the convergence of metabolism and immune modulation represents a promising avenue in the advancement of cancer immunotherapies [8].

Pyrimidine metabolism (PyM) orchestrates the synthesis, catabolism, and utilization of essential pyrimidine nucleobases, such as cytosine and uracil, integral to nucleic acid structure [9]. PyM's significance extends to nucleic acid synthesis and broader energy metabolism. Biosynthetic and degradation pathways involve de novo and salvage mechanisms, crucial in rapidly dividing cells [10]. Disruptions in PyM lead to a spectrum of inherited disorders, including autoimmune inflammatory conditions like AS. Contemporary studies reveal that miRNAs, like MiR-146a and miR-155, enhance AS proliferation by repressing genes inhibiting cellular proliferation [11]. TSHR-mediated modulation of miR-146a and miR-155 may shed light on AS fibroproliferative pathology. In autoimmune pathologies, Madera-Salcedo et al. [12]. demonstrate PPP2R2B (B55ß) dysregulation, induced by inflammation-driven hypermethylation, confers resistance to cytokine withdrawal-induced apoptosis [13]. Zhu et al. reveal UBE2T amplifies hepatocellular carcinoma progression, correlating with enhanced PyM [14]. This study aims to dissect PyMGs and their role in AS immunotherapy, exploring purinosome formation and glutamine PyM pathways for potential therapeutic avenues. Despite advances, the impact of PyM on the immunogenic landscape and its role in governing immunotherapeutic efficacy in AS remain unclear. This study seeks a holistic evaluation of PyMGs, unraveling their interplay with immunotherapy in AS, paving the way for groundbreaking clinical innovations.

**Fig. 1:**
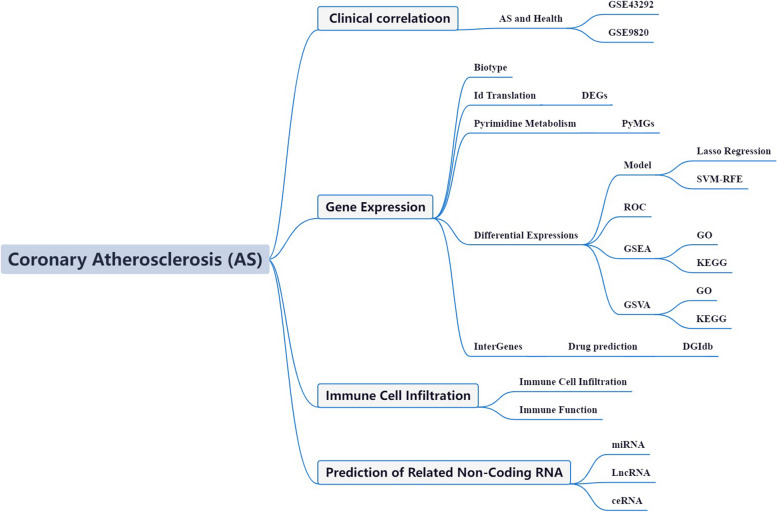
Framework

In advancing the frontier of AS research, our initiative employs high-throughput transcriptome sequencing and integrates it with detailed clinical data, marking a transformative phase in the study of this cardiovascular pathology. This methodology is pivotal in dissecting the transcriptional and molecular complexities intrinsic to AS. Our bioinformatics examination of these data has provided pivotal insights, substantially enriching our understanding of AS's pathophysiological mechanisms. Despite progress, the involvement of PyMGs in AS pathology remains largely uncharted. Our study is meticulously tailored to bridge this research void, harnessing the extensive AS-related data available in the GEO. We aim to decode the roles and impacts of PyMGs within the pathogenetic framework of AS. Figure 1 concisely illustrates our methodological approach and anticipated results, guiding through a thorough analytical process.

## Materials and Methods

The methodologies proposed by Zi-Xuan Wu et al. in 2023 were employed in this study [15].

### Raw Data

The GEO datasets GSE43292 and GSE9820 were utilized in this study. The platform used was GPL6244 and GPL6255. GSE43292 served as the training group, while GSE9820 served as the test group (Table 1). MSigDB included 105 PyMGs (Table.S1).

**Table 1:** The clinical characteristics of patients

GSE43292		GSE9820	
Variables	Number of samples	Variables	Number of samples
Gender		Gender	
Male/Female	unknown	Male/Female	64/89
Diagnosis		Diagnosis	
AS/Not-AS	32/32	AS/Not-AS	87/66
Tissue		Tissue	
Macroscopically intact tissue/Atheroma plaque	32/32	Macroscopically intact tissue/Atheroma plaque	unknown
Country	France		Netherlands

### Transcriptomic profiling and identification of Differentially Expressed Genes (DEGs)

The alignment and sorting of transcription data and human configuration files were meticulously executed using Perl, ensuring precision in acquiring mRNA gene expression data. Subsequent to data standardization from GSE43292, differential expression analysis ensued, employing stringent criteria of FDR < 0.05 and |log2FC|≥ 1, facilitated by the limma package. Pearson's correlation coefficient was judiciously applied for the identification of statistically significant and highly correlated genes within modules, employing the correlation analysis tool provided by the corrplot package.

### Functional annotation and pathway analysis

Functional annotation and pathway exploration for the identified DEGs were conducted using GO and KEGG analyses. R software, in conjunction with clusterProfiler, org.Hs.eg.db, enrichplot, and ggplot2 packages, was employed to decipher the impact of differentially expressed PyMGs on Biological Processes (BP), Molecular Functions (MF), and Cellular Components (CC), utilizing KEGG data.

### Model construction and immune cell infiltration analysis

In this research, a trio of machine learning algorithms—LASSO, Random Forest, and SVM-RFE —were adeptly utilized to identify key genetic markers. LASSO, known for its proficiency in dimension reduction, outperforms traditional regression analysis in handling high-dimensional data. This algorithm was fine-tuned using a penalty parameter and validated through a tenfold cross-validation process implemented via the glmnet package. The study further incorporated the RFE technique from the Random Forest algorithm, a supervised machine learning approach, to rank genes associated with atherosclerotic plaque progression and immune responses [16]. The effectiveness of this method was gauged through ten-fold cross-validation, pinpointing genes with a relative importance exceeding 0.25 as characteristic genes. SVM-RFE, distinguished for its ability to select relevant features while excluding redundant ones, proved more effective than linear discriminant analysis and the mean squared error method. This approach was similarly applied for feature selection, employing a ten-fold cross-validation strategys [17]. To evaluate the diagnostic accuracy of these methodologies, curves and the corresponding Area Under the Curve were used, providing a robust measure of the algorithms’ predictive capabilities in the context of genetic biomarker identification.

For the construction of predictive models, the glmnet package facilitated Lasso regression, augmented by cross-validation to enhance accuracy and reliability. Additionally, the support vector machine recursive feature elimination (SVM-RFE) algorithm, utilizing the e1071 package, was employed to craft a sophisticated machine learning model. Support vector machines (SVMs), renowned for their robustness in generalized linear classification, were leveraged for binary classification tasks. SVMs integrate the hinge loss function to assess empirical risk, and regularization terms confer sparsity and resilience, optimizing structural risk. Kernel methods were ingeniously applied to transcend linearity, establishing SVMs as exemplars in kernel learning techniques. Model robustness was evaluated through cross-validation, assessing error rates and predictive accuracy. The Lasso and SVM algorithms played a pivotal role in ranking the significance of feature genes. Decoding the composition of immune cells within samples was achieved using the CIBERSORT algorithm.

### Interrogating functional perturbations: gene set enrichment and variation analysis

Gene Set Enrichment Analysis (GSEA) and Gene Set Variation Analysis (GSVA) played pivotal roles in elucidating functional perturbations and pathway aberrations across diverse samples. Leveraging associated scores and visual representations, we scrutinized dynamic biological activities and pathways within discrete risk strata. Employing the R programming environment and a suite of packages, including limma, org.Hs.eg.db, clusterProfiler, and enrichplot, we delved into the impact of differentially expressed PyMGs on BP, MF, and CC, along with their intricate pathway involvements.

### Navigating the drug-gene nexus for precision therapeutics

In the epoch of bioinformatics ascendancy, the quest for efficacious biomarkers for disease diagnosis has intensified. Beyond discovery, the imperative lies in the practical application of these biomarkers in clinical scenarios. Predictive analytics for drug responses, informed by these biomarkers, stands as a cornerstone for advancing prevention and treatment modalities for AS. Validated biomarkers serve as navigational beacons guiding targeted therapeutic interventions. Precision in drug-gene interaction predictions is paramount, and in this study, we utilized the Drug-Gene Interaction database (DGIdb) (https://dgidb.genome.wustl.edu/) to forecast potential drug interactions with our identified hub genes.

### Unraveling the interplay of Non-coding RNAs: miRNAs and lncRNAs

The regulatory landscape of genetic expression is intricately shaped by non-coding RNA transcripts, including microRNAs (miRNAs) and long non-coding RNAs (lncRNAs). MiRNAs finely modulate gene expression by orchestrating mRNA degradation and translation, while lncRNAs, spanning around 200 nucleotides, govern an array of cellular physiological and biochemical pathways. Emerging research has unveiled a complex interplay between miRNAs and lncRNAs, featuring competitive binding dynamics among these molecules and other regulatory entities. The concept of competitive endogenous RNAs (ceRNAs) has emerged, wherein lncRNAs act as molecular sponges for miRNAs. Our study aims to decode specific miRNAs and lncRNAs sharing regulatory axes and developmental trajectories in AS, employing Perl software for this exploration.

### Constructing the multi-layered regulatory network: mRNA-miRNA-lncRNA interactions

Target gene data for common miRNAs and lncRNAs were sourced from empirically validated databases, including miRTarBase [18] and PrognoScan [19]. The regulatory network was meticulously constructed by mapping the intersections between the target genes of mRNA-miRNA-lncRNA and the genes implicated in AS. Visualization and analysis of this intricate network were facilitated using Cytoscape [20] software, offering a comprehensive view of the regulatory interactions at play.

### Statistical analysis

To delineate the diagnostic precision of the proposed biomarker candidate genes in the context of the pathological state, a comprehensive ROC analysis was executed utilizing ROCplotter. This approach focused on evaluating the specificity and sensitivity of these biomarkers, with the AUC metric serving as the critical indicator of biomarker efficacy. Here, the AUC value is posited as a direct reflection of biomarker quality, adhering to the principle that higher values denote superior biomarker performance [21]. Further, to ascertain the statistical significance of variations in expression levels of the candidate genes under conditions with and without AS, the non-parametric Mann–Whitney test was employed. This test provided a robust framework for evaluating disparities in gene expression profiles pertinent to the AS condition. Additionally, a Spearman correlation analysis was conducted. This analysis was pivotal in establishing a relationship between the log2-transformed expression values of the candidate genes and the log2-transformed expression values of canonical markers of AS [22]. Such a correlation study is instrumental in reinforcing the biological relevance of the candidate genes within the AS pathological framework, thereby enriching the validity of these biomarkers in the context of AS diagnosis and prognosis.

## Results

### Identification of DEGs and principal component analysis

**Fig. 2:**
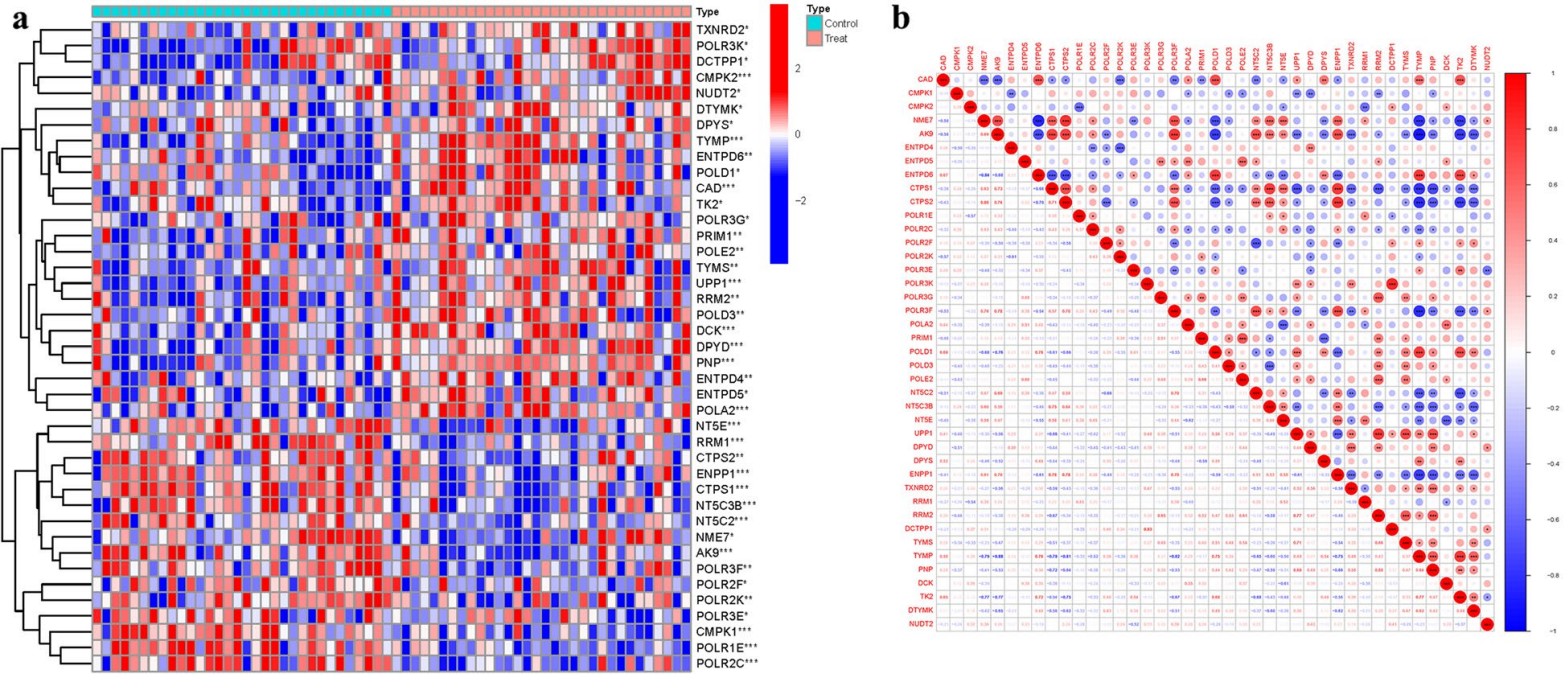
Principal Component Analysis. **a** Analysis of difference. **b** Analysis of correlation

Among the 41 examined PyMGs, several exhibited significant differences in expression levels. Furthermore, gene clustering analysis revealed distinct clusters in the treatment and control groups. Notable PyMGs in the treatment group included TXNRD2, POLR3K, DCTPP1, CMPK2, NUDT2, DTYMK, DPYS, TYMP, ENTPD6, while the control group included NT5E, RRM1, CTPS2, ENPP1, CTPS1, NT5C3B, NT5C2, NME7, etc. (Fig. 2a). Correlation analysis was conducted among these PyMGs, and a correlation matrix was generated for visualization (Fig. 2b) (Table S2).

### Enrichment analysis of PyMGs

**Fig. 3:**
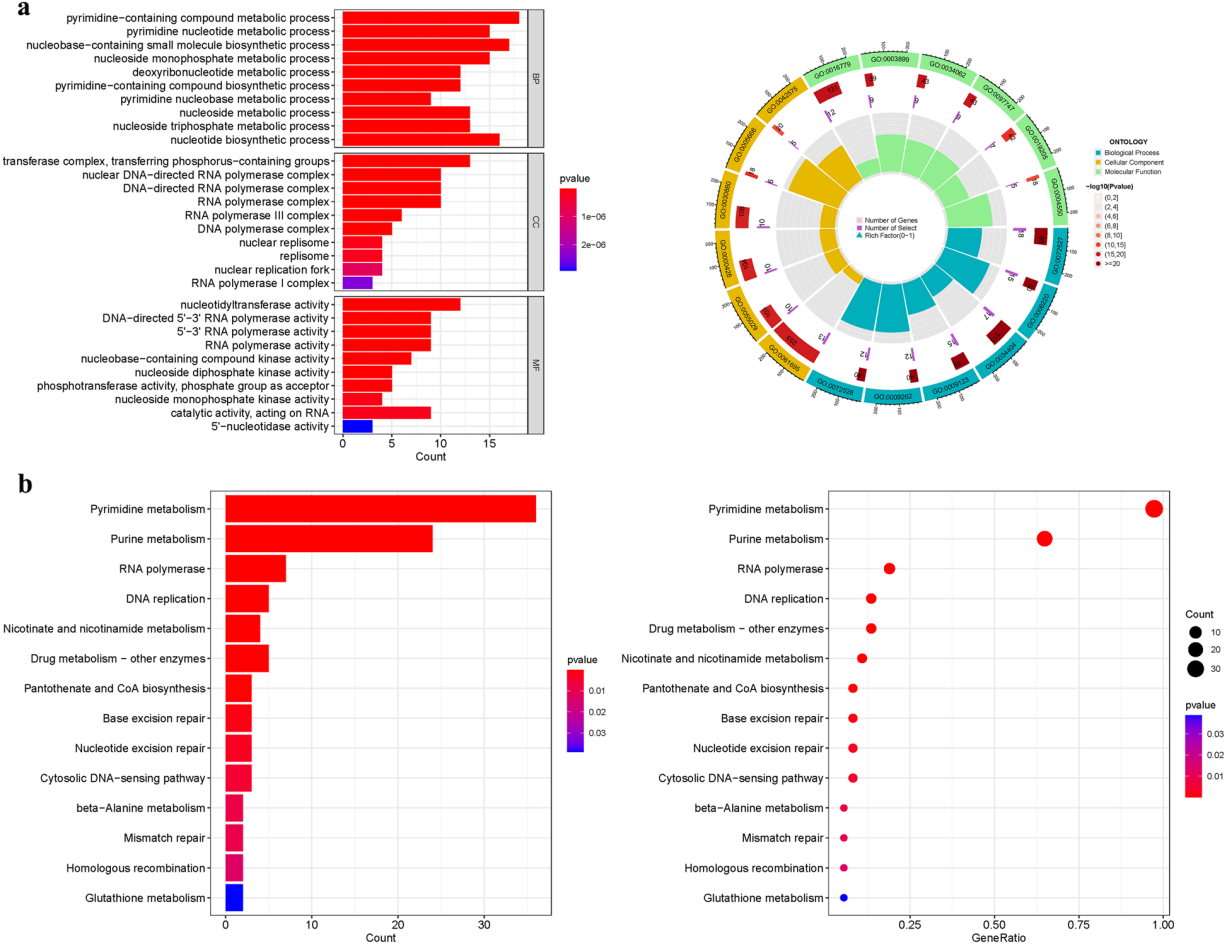
For PyMGs, GO, and KEGG analyses were performed. **a** The GO circle illustrates the scatter map of the selected gene's logFC. **b** The KEGG barplot and bubble illustrates the scatter map of the logFC of the indicated gene

GO enrichment analysis identified 299 core target genes, encompassing BP, MF, and CC. The MF category primarily involved nucleotidyltransferase activity (GO:0016779), DNA-directed 5'-3' RNA polymerase activity (GO:0003899), 5'-3' RNA polymerase activity (GO:0034062). The CC category was mainly associated with transferase complex, transferring phosphorus-containing groups (GO:0061695), nuclear DNA-directed RNA polymerase complex (GO:0055029), DNA-directed RNA polymerase complex (GO:0000428). The BP category included pyrimidine-containing compound metabolic process (GO:0072527), nucleobase-containing small molecule biosynthetic process (GO:0034404), nucleotide biosynthetic process (GO:0009165). KEGG enrichment analysis revealed that the upregulated genes were primarily involved in RNA polymerase (hsa03020), Pyrimidine metabolism (hsa00240), hsa00230 (Purine metabolism) (Fig. 3 and Table S3a-b).

### Model construction

**Fig. 4:**
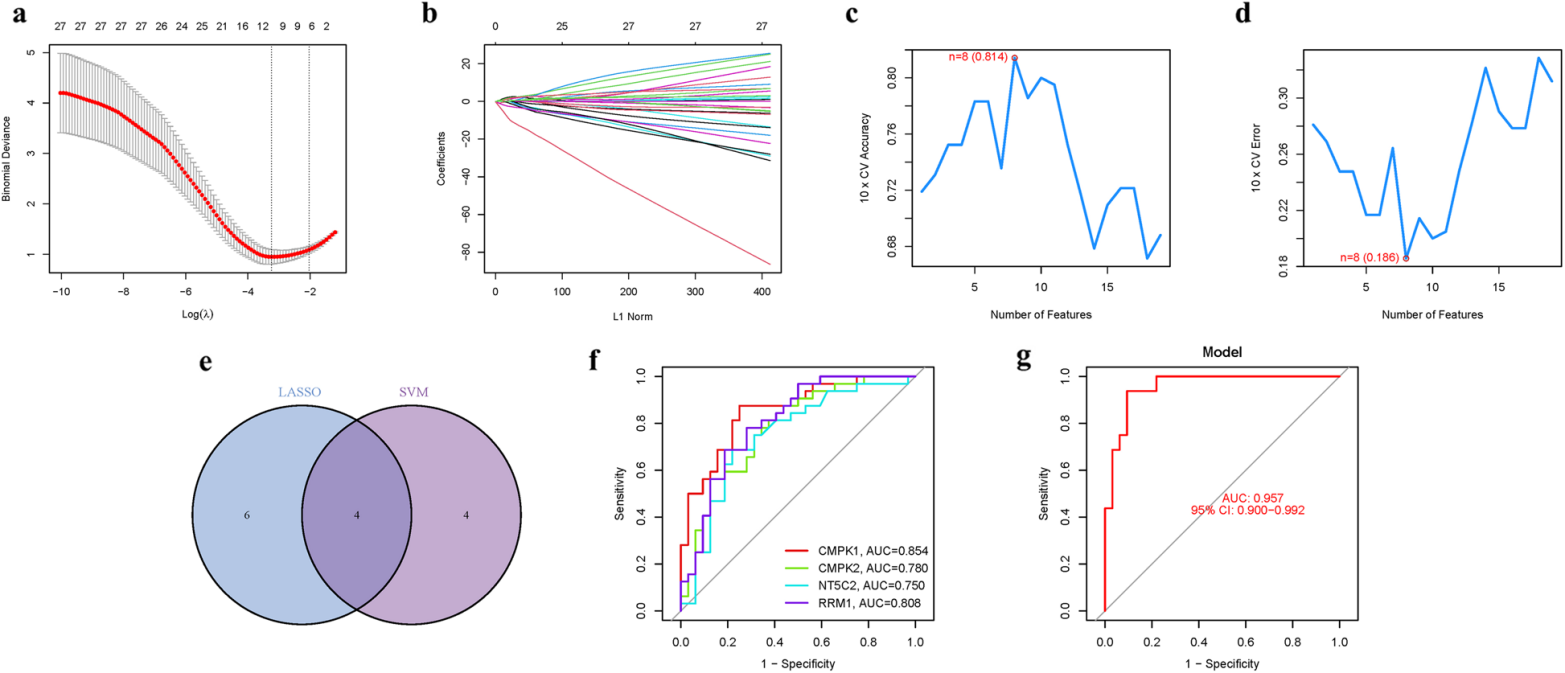
The development of the PyMGs signature. **a** Regression of the 4 AS-related genes using LASSO. **b** Cross-validation is used in the LASSO regression to fine-tune parameter selection. **c**-**d** Accuracy and error of this model. **e** Venn. **f** AUC of 4 hub genes. **g** AUC of train group

In our study, we developed a robust gene signature by integrating LASSO (Least Absolute Shrinkage and Selection Operator) and Cox regression analyses, with optimization detailed in Fig. 4a-b. The model's fidelity was rigorously tested through Support Vector Machine—Recursive Feature Elimination (SVM-RFE), demonstrating an accuracy rate of 0.814, with an error margin of 0.186, as illustrated in Fig. 4c-d. A comparative analysis between the four Pyrimidine Metabolism Genes (PyMGs) identified via LASSO and SVM-RFE exhibited significant concordance, thereby validating the integrity of our model (Fig. 4e). When applied specifically to the four hub genes—CMPK1, CMPK2, NT5C2, and RRM1—the model exhibited high precision, with Area Under Curve (AUC) values of 0.854, 0.780, 0.750, and 0.808, respectively (Fig. 4f). Remarkably, within the dataset GSE9820, our model achieved an AUC of 0.957 (95% CI 0.900–0.992), underpinning its accuracy and robust predictive capability (Fig. 4g) (Table 2 and S4). The performance metrics, particularly the AUC, as prominently displayed in Fig. 4, substantiate the model’s precision. The AUC value of 0.957 notably highlights this precision. It's important to consider that potential variations in AUC may result from genetic heterogeneity among individuals. However, it is crucial to note that the collective AUC values for the implicated genes consistently approximate or surpass the 0.7 threshold, indicative of a strong predictive performance. This synthesis of results bolsters the credibility and robustness of our model, affirming its potential utility in predictive diagnostics within the context of PyMGs.

**Table 2:** The characteristics of model

Label	LASSO	SVM-RFE
Sensitivity	1.000000	0.916667
Specificity	0.692308	0.769231
Pos Pred Value	0.750000	0.785714
Neg Pred Value	1.000000	0.909091
Precision	0.750000	0.785714
Recall	1.000000	0.916667
F1	0.857143	0.846154
Prevalence	0.480000	0.480000
Detection Rate	0.480000	0.440000
Detection Prevalence	0.640000	0.560000
Balanced Accuracy	0.846154	0.842949

### Gene set enrichment analysis

**Fig. 5:**
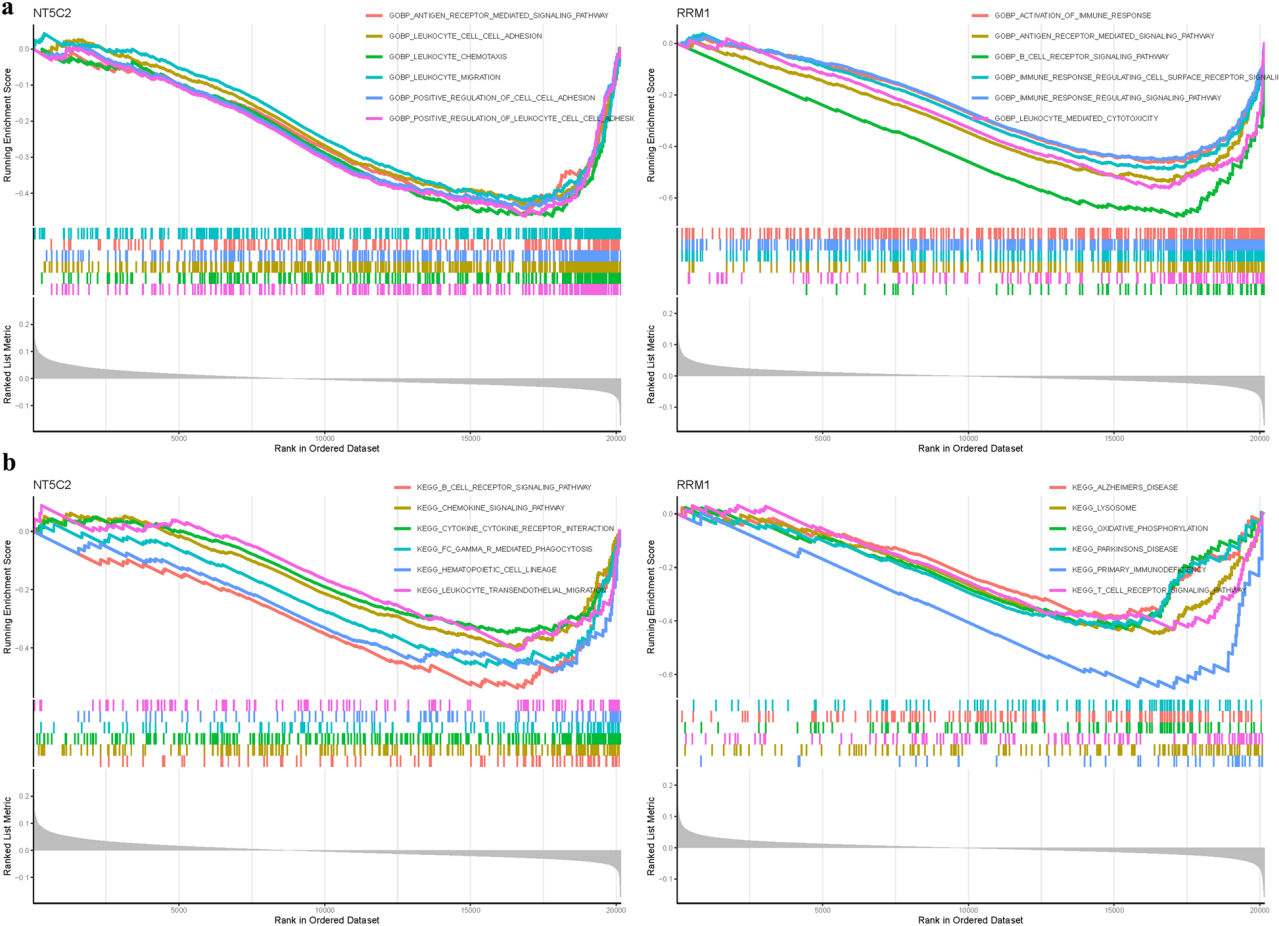
GSEA of Analysis in NT5C2 and RRM1. **a** GO. **b** KEGG

In this study, the AUC of each gene in the test group, the Rank ranking of each gene, and the results of the test group validation were observed. We found that NT5C2 and RRM1 may be the most relevant genes. Through literature evaluation and analysis of hub gene sensitivity within the model, it was determined that NT5C2 and RRM1 may be the most relevant genes to AS. In terms of GO analysis, NT5C2 was found to be associated with BP leukocyte chemotaxis, BP positive regulation of leukocyte cell cell adhesio, BP positive regulation of cell cell adhesion. On the other hand, RRM1 was primarily involved in the BP b cell receptor signaling pathway, BP antigen receptor mediated signaling pathway, BP leukocyte mediated cytotoxicity (Fig. 5a). In KEGG analysis, NT5C2 was mainly associated with KEGG b cell receptor signaling pathway, KEGG cytokine cytokine receptor interaction, KEGG chemokine signaling pathway, while RRM1 was involved in KEGG primary immunodeficiency, KEGG oxidative phosphorylation, KEGG parkinsons disease (Fig. 5b) (Table S5).

### Analysis of immune cells

**Fig. 6:**
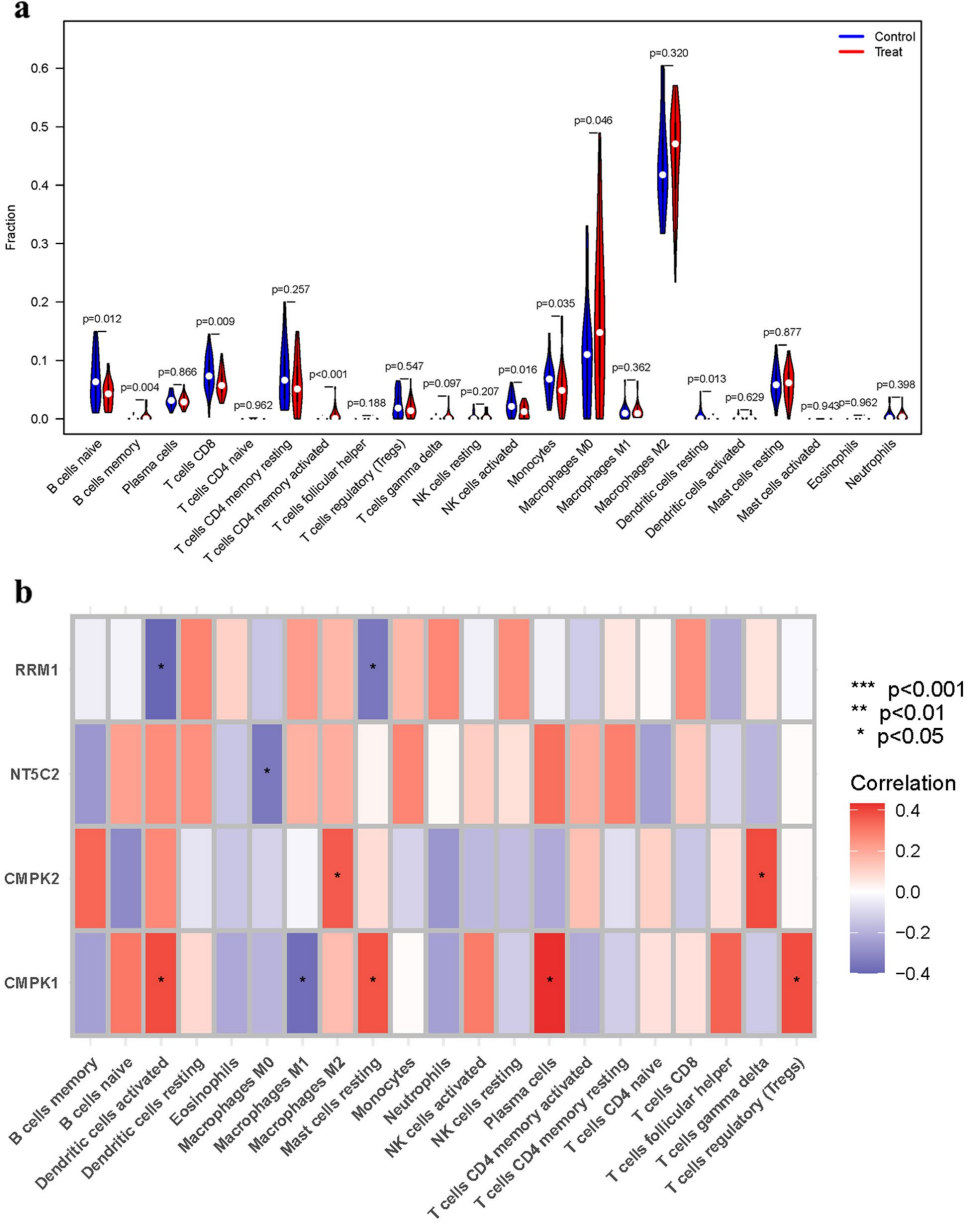
Expression of Immune cells. **a** Expression of immune cells in different clusters. **b** Correlation between PyMGs and immune cells

The immune microenvironment plays a crucial role in the initiation and progression of AS. A vioplot was created to display the expression patterns of B cells naive, T cells CD8, NK cells activated, Monocytes, Dendritic cells resting, which were highly expressed in the control group. While, B cells memory, T cells CD4 memory activated, Macrophages M0 were highly expressed in the treatment group (Fig. 6a). Additionally, a correlation analysis was performed to investigate the relationship between these genes and immune cells (Fig. 6b).

### GSVA

**Fig. 7:**
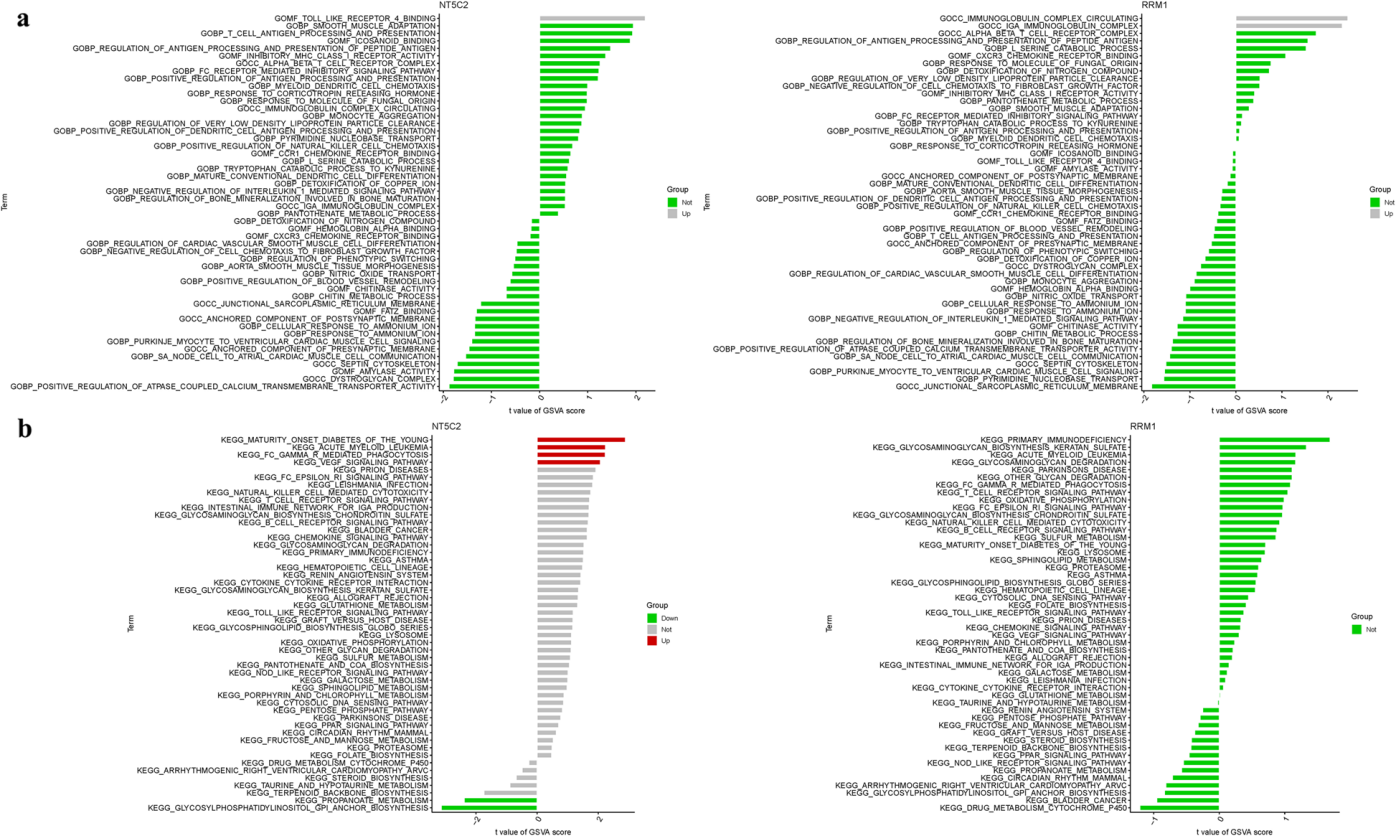
GSVA of Analysis in NT5C2 and RRM1. **a** GO. **b** KEGG

In the GO analysis, NT5C2 was primarily associated with BP regulation of antigen processing and presentation of peptide antigen, MF icosanoid binding, BP t cell antigen processing and presentation, BP smooth muscle adaptation, MF toll like receptor 4 binding. RRM1 was mainly involved in the MF cxcr3 chemokine receptor binding, BP l serine catabolic process, BP regulation of antigen processing and presentation of peptide antigen, CC alpha beta t cell receptor complex, CC iga immunoglobulin complex, CC immunoglobulin complex circulating (Fig. 7a). In terms of KEGG analysis, NT5C2 was mainly associated with prion diseases, vegf signaling pathway, fc gamma r mediated phagocytosis, acute myeloid leukemia, maturity onset diabetes of the young. RRM1 was involved in parkinsons disease, glycosaminoglycan degradation, acute myeloid leukemia, glycosaminoglycan biosynthesis keratan sulfate, primary immunodeficiency (Fig. 7b).

### Drug-gene interactions

**Fig. 8:**
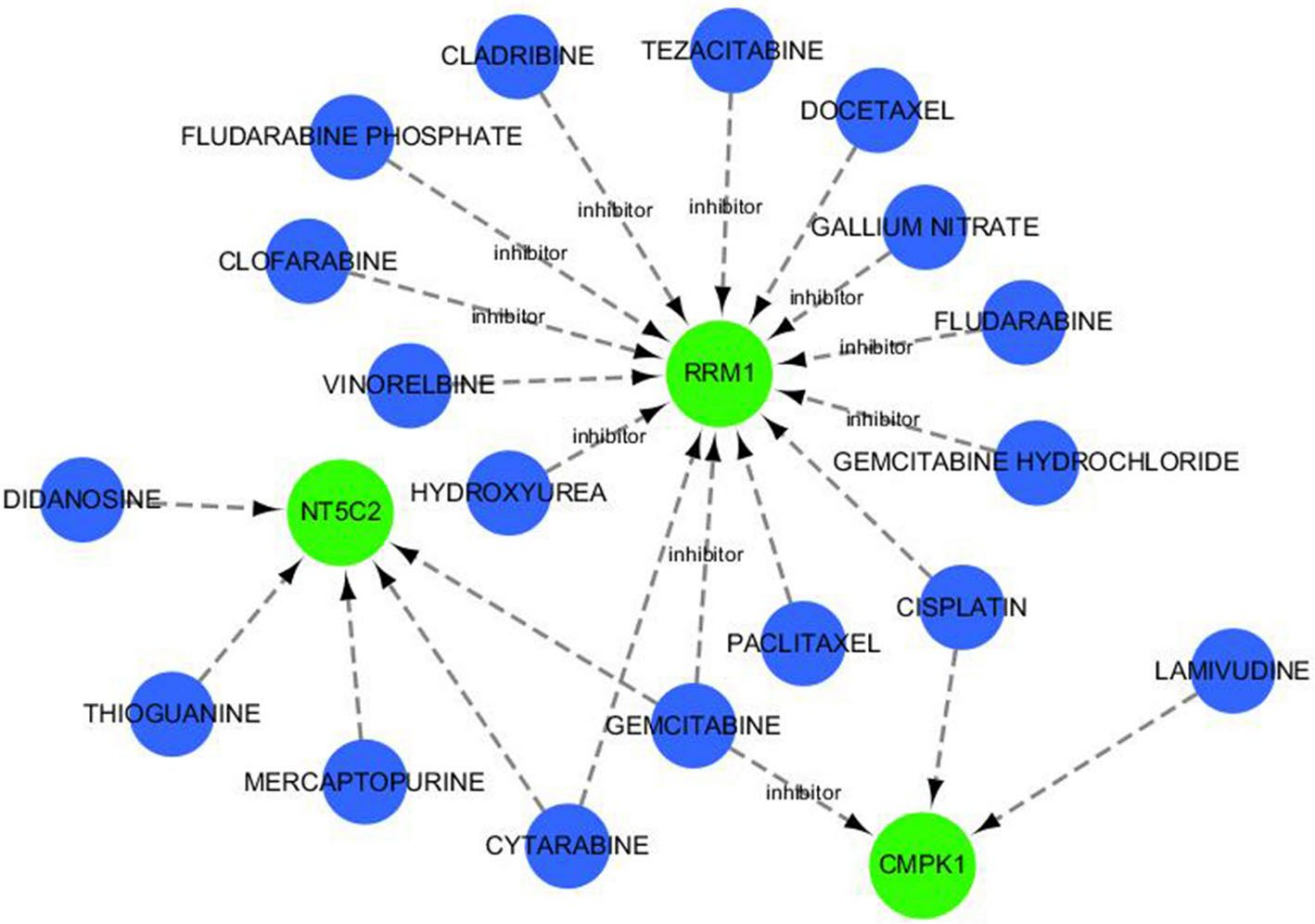
Drug-gene interactions. Note: Red circles are up-regulated genes, green hexagons are down-regulated genes, and blue squares are associated drugs

Three drugs were predicted to interact with the hub genes, including gemcitabine, lamivudine, cisplatin, didanosine, gemcitabine, mercaptopurine (Table S6) (Fig. 8).

### Identification of common RNAs and construction of miRNA-lncRNA shared genes network

**Fig. 9:**
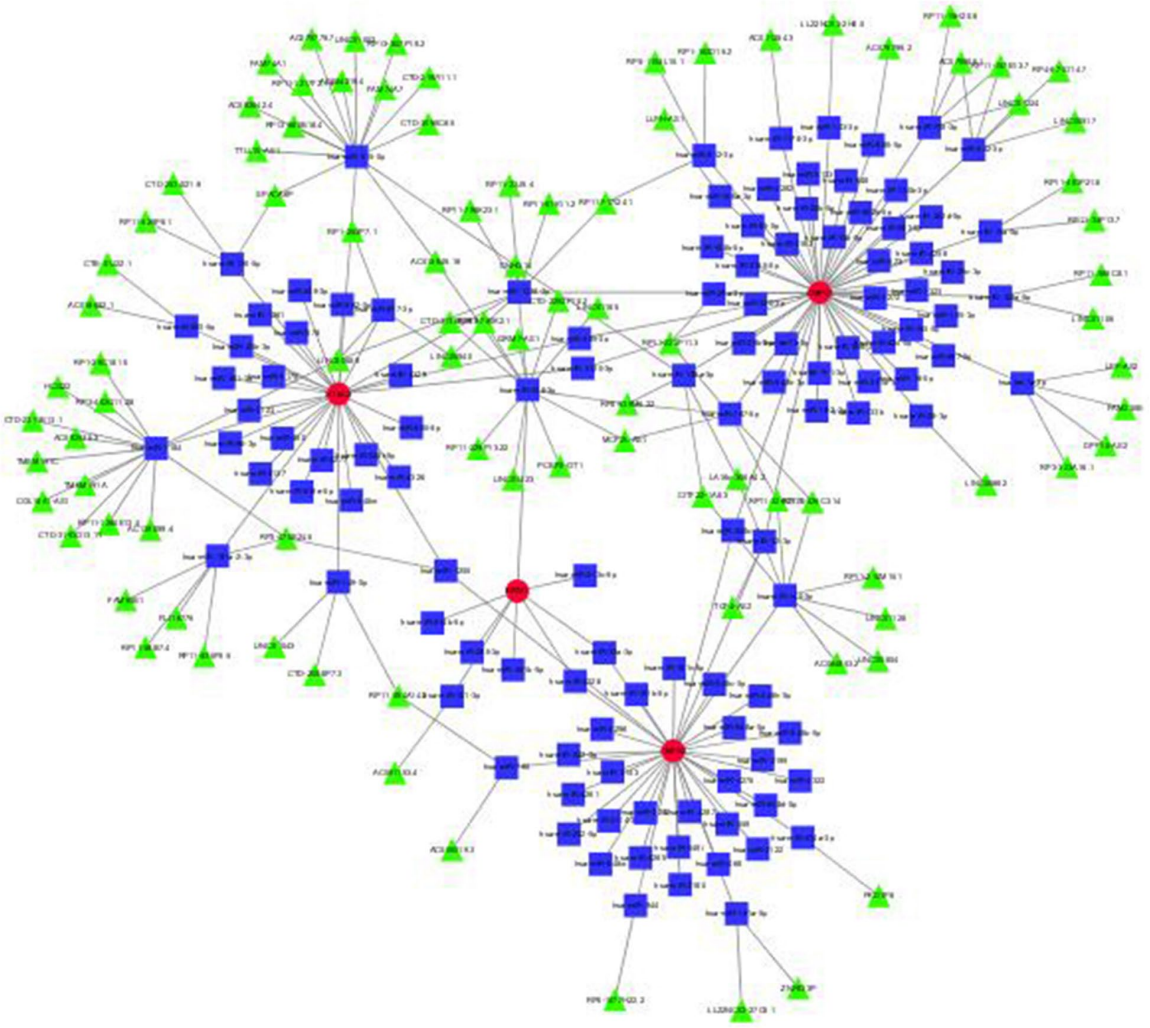
miRNAs-LncRNAs shared Genes Network. Note: Red circles are mrnas, blue quadrangles are miRNAs, and green triangles are lncRNAs

A total of 123 miRNAs and 114 lncRNAs associated with AS were identified from three databases (Table S7a-b). Table S7 shows the matching of these genes against the corresponding miRNA database. These databases include miRanda [23], miRDB [24], and TargetScan [25]. When the corresponding database matched the relevant miRNA, the score was marked as 1. It can be seen that when all three databases can be matched, it is 3 points. The miRNA was matched by spongeScan database [26] to obtain the corresponding lncRNA data. The miRNA-lncRNA-gene network was constructed by intersecting these non-coding RNAs with the shared genes obtained through Lasso regression and SVM-RFE. The network consisted of 92 lncRNAs, 115 miRNAs, and some common genes, including the four hub genes (CMPK2, CMPK1, NT5C2, and RRM1) (Fig. 9). In this method, a network is constructed by matching the relevant data sets and matching the mirnas with higher scores with related genes, etc., through the scoring file. This resulted in some less relevant ones being eliminated during the screening process.

### Validation of hub genes and model

**Fig. 10:**
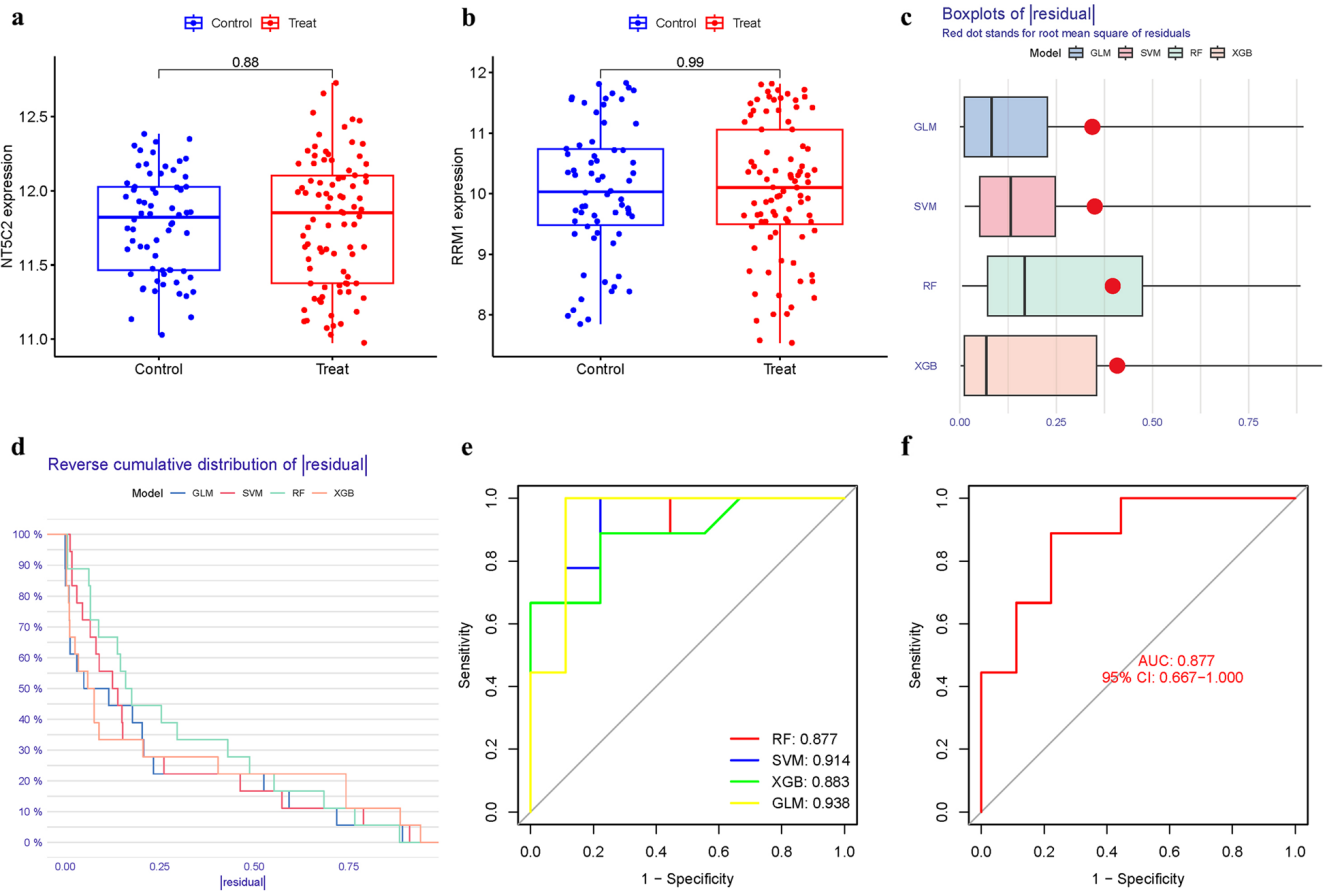
Hub gene and Model verification. **a**-**b** Hub genes were validated. **c**-**d** Residual expression patterns. **e** AUC of model. **f** AUC of GSE9820 group

To enhance the confidence and prediction accuracy of the model, GSE105149 dataset was used for validation. The GSE105149 analysis further confirming their potential relevance to AS (Fig. 10a-b). The Boxplots depicted the residual expression patterns of these genes in AS (Fig. 10c-d). In this model, which positions four GlnMgs at the forefront, NT5C2 and RRM1 were hypothesized to exhibit significant disparities. Contrary to expectations, as delineated in the figure, these genes did not attain statistical significance. This outcome is likely attributable to limitations in sample size and regional ethnic variations. Nonetheless, the uniformity of these results across independent datasets bolsters the reliability of these biomarkers, reinforcing their potential in elucidating the molecular framework of AS. This consistency, despite initial results, highlights the nuanced complexity of AS's molecular landscape and the importance of robust, diverse datasets in genetic research. The PyMGs' diagnostic capacity in distinguishing AS from control samples revealed a satisfactory diagnostic value, with an AUC of RF: 0.877; SVM: 0.914; XGB: 0.883; GLM: 0.938 (Fig. 10e). An AUC of 0.877 (95% CI 0.667–1.000) in GSE9820 (Fig. 10f).

## Discussions

Atherosclerosis, a covert architect of vascular pathology, steadfastly remains a leading precursor to cardiovascular morbidity and mortality. Within this paradigm, the need for advanced diagnostic approaches to effectively stratify atherosclerotic risk is pressing. The evolution of atherosclerotic plaques is a multifaceted process, bearing resemblance to the immune intricacies characteristic of oncological disorders. It is therefore critical to gain a profound understanding of the roles played by immune-associated genes in the development of these plaques. Mirroring the metabolic upheavals characteristic of cancer, where aberrant glycolysis serves as a hallmark of malignancy,metabolic markers have gained prominence [27]. Markers such as those involved in cysteine and nucleotide metabolism, alongside oncometabolites like 2-hydroxyglutarate, have shown promise in the diagnostic and therapeutic arenas of glioma. In this milieu, gene expression regulation assumes a pivotal role [28]. Research centering on PyM has sought to elucidate the intricate ties between metabolic dysregulation and inherent genetic variances within the cancerous milieu [29]. Studies have identified heightened pyrimidine synthesis flux in cells harboring mutations in KRAS, PTEN, or p53—rendering them vulnerable to targeted synthetic lethality approaches [30]. Inhibition of the pyrimidine biosynthetic pathway thus stands as a potential novel therapeutic avenue for tumors harboring such gain-of-function mutations [31]. The interplay between the pyrimidine pathway and other metabolic routes provides an expansive understanding of metabolic heterogeneity, paving the path for the development of individualized therapies. Contemporary advances in oncological research have redirected attention from the tumor per se to encompass a holistic grasp of non-cancerous biological processes [31]. From such a vantage point, the investigation into distinct PyM signatures during the progression of atherosclerosis harbors significant potential. Probing the diverse PyM patterns along the continuum of atherosclerotic progression offers invaluable insights into the role of PyM in the pathogenesis of atherosclerosis and holds the key to unlocking novel targeted therapeutic strategies.

In this comprehensive study, we have elucidated a group of 41 DEGs intimately linked to glutamine metabolism in the context of AS. By integrating DEGs with advanced analytical techniques like Lasso regression and SVM-RFE, we identified four pivotal PyMGs—CMPK1, CMPK2, NT5C2, and RRM1. These genes emerged as significant, showing promising diagnostic potential, a conclusion reinforced through external dataset validation. This suggests their integral role in the etiology of AS. This research has elucidated the potential significance of drug-gene interactions and non-coding RNA networks in highlighting the relevance of four marker genes associated with AS. Through our analysis, we identified seven key genes (CMPK2, CMPK1, NT5C2, and RRM1) that are intricately associated with the progression of atherosclerotic plaques and immune responses. These genes hold promise for predicting the advancement of atherosclerotic plaques. Additionally, our study proposes a novel molecular classification that differentiates between immune and non-immune subtypes within atherosclerotic plaques. This classification could have substantial implications in the realm of cardiovascular diseases. Overall, our findings pave the way for the development of more precise and individualized cardiovascular immunotherapies, potentially revolutionizing treatment approaches in this field. Our review of the literature highlighted NT5C2 and RRM1 as key players in AS association. Further analysis of their biological functions revealed their involvement in the metabolism of pyrimidine-containing compounds, the biosynthesis of nucleobase-containing small molecules, and nucleotide biosynthesis. These findings position PyMGs as central to a range of biological pathways, likely influencing immune-related processes and thus significantly impacting the pathophysiology of AS.

RRM1, integral to the rate-limiting enzyme in nucleotide synthesis, emerges as a prospective prognostic biomarker for advanced non-small cell lung cancer (NSCLC). Its elevated expression is linked to reduced efficacy of certain chemotherapeutics, underscoring its clinical relevance. Numerous studies have highlighted the influence of RRM1 levels on NSCLC prognosis [32]. In a significant development, Reglero et al. identified CRCD2 as an innovative small molecule inhibitor targeting NT5C2 nucleotidase, exhibiting potent efficacy against common mutant forms implicated in leukemia relapse, both in vitro and in vivo. Notably, CRCD2 enhances the cytotoxicity of 6-MP in leukemias with wild type NT5C2, revealing NT5C2 S502 phosphorylation as a novel resistance mechanism to mercaptopurine [33]. Complementary research by Lai emphasizes CMPK2's role in regulating DENV-induced cytokine release, mitochondrial oxidative stress, and mitochondrial DNA translocation. The study demonstrates that CMPK2 depletion reduces DENV-induced Toll-like receptor (TLR)-9 activation, inflammasome pathway engagement, and cell migration, albeit with increased viral proliferation [34]. Similarly, Chen's research in a spinal cord injury (SCI) model shows that CMPK2 influences NLRP3 expression, which is crucial in inflammasome activation and subsequent inflammation post-SCI. Intriguingly, electroacupuncture reduces CMPK2 expression and NLRP3 activation, enhancing motor function recovery in SCI rats [35]. These studies collectively underline the importance of PyMGs, including NT5C2 and RRM1, in AS as examined in our research. Data from the GSE9820 dataset further suggest PyM-related features as potential robust prognostic markers. However, the exploration into gene alterations linked to PyM is in its infancy, necessitating more comprehensive research to fully grasp their clinical implications.

Atherosclerosis, a chronic inflammatory disorder with immune underpinnings, represents a perilous journey towards cardiovascular disaster, characterized by the relentless formation of arterial intimal plaques. This pathophysiological odyssey, rooted in early inflammation, culminates in the complex evolution and rupture of atherosclerotic plaques [36]. A diverse array of immune cells -including monocytes/macrophages, T lymphocytes, dendritic cells, and mast cells-plays a pivotal role in orchestrating atherosclerosis progression through their dynamic interactions. Within the arterial milieu, monocytes transform into macrophages, initiating lipid accumulation and foam cell formation, quintessential features of atherosclerosism [37]. CD4 + T cells, as key inflammatory players, exacerbate the inflammatory environment and contribute to the array of pro-inflammatory cytokines. Dendritic cells, at the interface of innate and adaptive immunity, finely tune the immune response within the developing lesion [38]. Mast cells, on the other hand, release inflammatory mediators that heighten inflammation and promote plaque instability.

The intricate interplay among immune and vascular cells is governed by a complex network of cytokines, chemokines, and inflammatory signals. Pro-inflammatory cytokines, such as IL-1β, IL-6, and TNF-α, serve as harbingers of endothelial activation and plaque progression [39]. Chemokines recruit immune cells to the inflammation site, intensifying its magnitude. Additionally, the surveillance roles of Toll-like receptors (TLRs) and NOD-like receptors (NLRs), which detect pathogen-associated and damage-associated molecular patterns, initiate the atherosclerotic inflammatory cascade [40]. Deciphering this immunological web has expanded our understanding of atherosclerosis and revealed new therapeutic targets. Immunomodulatory strategies focusing on specific immune cell subsets or inflammatory pathways offer a revolutionary approach in preventing and stabilizing atherosclerotic plaques, potentially diminishing cardiovascular events [41]. Building upon previous research, our study delves into the expression of PyMGs within the atherosclerosis immune milieu. Using vioplot visualization, we discerned the expression patterns of various immune cells. Naive B cells, CD8 T cells, activated NK cells, monocytes, and resting dendritic cells predominated in the control group. In contrast, memory B cells, activated memory CD4 T cells, and M0 macrophages were notably expressed in the treatment group, indicating a shift in the immune landscape influenced by therapeutic intervention.

The emerging field of research exploring the complex relationship between AS and metabolic dynamics is gaining momentum, particularly with the advancements in bioinformatics [42–44]. Notable contributions in this domain include Zemin Tian’s work on immunogenic cell death in endothelial cells, and Shuangyang Mo’s exploration of molecular parallels between Non-Alcoholic Fatty Liver Disease (NAFLD) and AS. Additionally, Chi Ma’s predictive model, grounded in autophagy-related genes, offers promising avenues for diagnostic and therapeutic biomarkers. However, the association between purine metabolism and AS is still relatively uncharted, especially in terms of predictive models. Our study seeks to fill this void by employing an innovative methodology, leveraging extensive PyM datasets from the GEO, particularly GSE43292, with further validation through GSE9820. By integrating GO, KEGG analyses, and GSEA, our findings not only provide theoretical insights but also chart a course for future metabolic research and therapeutic strategies in AS. Despite these advancements, our research acknowledges certain limitations, such as the necessity for additional in vivo and in vitro experiments to fully unravel the complexities of AS mechanisms. The prognostic potential of PyMGs in the context of AS opens up a vista of opportunities for future investigation and innovation, indicating a significant scope for advancing understanding and treatment strategies in this field.

## Conclusions

The study of AS reveals a complex interplay of multiple elements, including a range of targets, signaling pathways, and regulatory mechanisms, with PyMGs such as CMPK1, CMPK2, NT5C2, and RRM1 at its core. Notably, NT5C2 and RRM1 emerge as critical regulators within this network. These genes are central to key metabolic processes, particularly in pyrimidine compound metabolism and nucleotide biosynthesis. The ability of NT5C2 and RRM1 to either stimulate or inhibit these pathways underscores their role in AS pathogenesis, reflecting the disease's metabolic flexibility. This insight into the dual functionality of these genes not only deepens our understanding of AS's molecular basis but also highlights the complex and dynamic nature of its pathobiology.

### Supplementary Information

**Supplementary Material 1. **

## Data Availability

The datasets generated and/or analysed during the current study are available in the [GEO] repository. https://www.ncbi.nlm.nih.gov/geo/ The datasets generated during and/or analyzed during the current study are available in the appendix.

## Abbreviations

AS: Atherosclerosis
GO: Gene Ontology
TCM: Traditional Chinese medicine
MF: Molecular functions
KEGG: Kyoto Encyclopedia of Genes and Genomes
GEO: Gene Expression Omnibus
PyMGs: Pyrimidine metabolism genes
BP: Biological processes
CC: Cellular components
DEGs: Differentially Expressed Genes
