# Supplementary material for: Unlocking potential biomarkers bridging coronary atherosclerosis and pyrimidine metabolism-associated genes through an integrated bioinformatics and machine learning approach
Source: BMC Cardiovasc Disord. 2024 Mar 7;24:148. doi: 10.1186/s12872-024-03819-w (PMC10921789; doi:10.1186/s12872-024-03819-w)
Supplement: Supplementary file 1 — Supplementary Material 1. [file 12872_2024_3819_MOESM1_ESM.doc]

Integrative Bioinformatics and Machine Learning Approach Unveils Potential Biomarkers Linking Coronary Atherosclerosis and Pyrimidine Metabolism-Associated Gene

**Supplementary appendix to the manuscript**

Contents of supplementary appendix

[Appendix 1 3](#__RefHeading___Toc9746)

[Datasets and Pyrimidine Metabolism genes 3](#__RefHeading___Toc9684)

[Table S1. Pyrimidine Metabolism genes 3](#__RefHeading___Toc4684)

[Appendix 2 4](#__RefHeading___Toc16475)

[DEGs linked to PyMGs 4](#__RefHeading___Toc30650)

[Table S2. 41 DEGs linked to PyMGs. 4](#__RefHeading___Toc31014)

[Appendix 3 6](#__RefHeading___Toc31161)

[Table S3a. Analysis of GO. 6](#__RefHeading___Toc14120)

[Table S3b. Analysis of KEGG. 23](#__RefHeading___Toc6225)

[Appendix 4 24](#__RefHeading___Toc25512)

[Table S4a. LASSO genes. 24](#__RefHeading___Toc17311)

[Table S4b. SVM-RFE genes. 24](#__RefHeading___Toc29299)

[Table S4c. InterGenes. 25](#__RefHeading___Toc31679)

[Appendix 5 25](#__RefHeading___Toc22706)

[Table 5a. NT5C2 of GSEA analysis. 25](#__RefHeading___Toc26825)

[Table 5b. RRM1 of GSEA analysis. 30](#__RefHeading___Toc20326)

[Appendix 6 33](#__RefHeading___Toc29654)

[Table 6. Drug prediction. 33](#__RefHeading___Toc26332)

[Appendix 7 35](#__RefHeading___Toc28983)

[Table 7a. Gene-miRNA. 35](#__RefHeading___Toc23185)

[Table 7b. Gene-lncRNA. 39](#__RefHeading___Toc11574)

# Appendix 1

**Datasets and Pyrimidine Metabolism genes**

**Table S1. Pyrimidine Metabolism genes**

| CAD | POLR1D | DCTD | ENPP1 | TWISTNB |
| --- | --- | --- | --- | --- |
| DHODH | POLR3H | TK2 | ENPP3 | ENTPD6 |
| UMPS | POLR3GL | TK1 | TXNRD1 | CTPS1 |
| CMPK1 | POLR3G | DTYMK | TXNRD2 | CTPS2 |
| CMPK2 | POLR3F | NUDT2 | TXNRD3 | POLR1A |
| NME6 | POLA1 | POLR2H | RRM1 | POLR1B |
| NME7 | POLA2 | POLR2I | RRM2B | ZNRD1 |
| NME2 | PRIM1 | POLR2L | RRM2 | POLR2C |
| NME4 | PRIM2 | POLR2J | DCTPP1 | POLR2D |
| NME1 | POLD1 | POLR2J3 | DUT | POLR2E |
| NME3 | POLD2 | POLR2J2 | TYMS | POLR2F |
| NME1-NME2 | POLD3 | POLR2K | CDA | POLR2G |
| AK9 | POLD4 | POLR3A | TYMP | POLR1E |
| PNPT1 | POLE | POLR3B | PNP | UPP1 |
| ENTPD3 | POLE2 | POLR3C | DCK | UPRT |
| ENTPD8 | POLE3 | POLR3D | NT5C2 | DPYD |
| ENTPD1 | POLE4 | POLR3E | NT5C1A | DPYS |
| CANT1 | UCK1 | RPC5 | NT5C1B | UPB1 |
| ENTPD4 | UCK2 | POLR1C | NT5C | POLR2A |
| ENTPD5 | UCKL1 | POLR3K | NT5M | NT5C3A |
| POLR2B | UPP2 | NT5E | NT5C1B-RDH14 | NT5C3B |

# Appendix 2

## **DEGs linked to PyMGs**

**Table S2. 41 DEGs linked to PyMGs.**

| Gene | conMean | treatMean | pvalue | Type |
| --- | --- | --- | --- | --- |
| CAD | 6.782762656 | 6.937931438 | 0.000728019 | Up |
| CMPK1 | 10.04166922 | 9.850467125 | 1.21E-06 | Down |
| CMPK2 | 6.950112031 | 7.223804219 | 7.16E-05 | Up |
| NME7 | 7.16382575 | 6.959316344 | 0.013036675 | Down |
| AK9 | 4.767765031 | 4.522629125 | 0.000246666 | Down |
| ENTPD4 | 8.439021844 | 8.542311563 | 0.003844702 | Up |
| ENTPD5 | 6.318437563 | 6.439430875 | 0.014648601 | Up |
| ENTPD6 | 7.479839688 | 7.704691344 | 0.003203989 | Up |
| CTPS1 | 8.805394813 | 8.233048031 | 1.06E-05 | Down |
| CTPS2 | 7.537726531 | 7.352287125 | 0.008494577 | Down |
| POLR1E | 7.2895955 | 6.841409406 | 2.49E-07 | Down |
| POLR2C | 8.076130875 | 7.834144313 | 4.46E-06 | Down |
| POLR2F | 7.9398045 | 7.873120438 | 0.048694339 | Down |
| POLR2K | 9.546122406 | 9.404553188 | 0.008663914 | Down |
| POLR3E | 7.497898719 | 7.425939156 | 0.038698555 | Down |
| POLR3K | 7.389706031 | 7.529538188 | 0.033801596 | Up |
| POLR3G | 5.132664313 | 5.264679313 | 0.027187345 | Up |
| POLR3F | 7.026185969 | 6.860365938 | 0.005245896 | Down |
| POLA2 | 6.664809031 | 6.874239031 | 8.70E-06 | Up |
| PRIM1 | 6.653324594 | 6.831997625 | 0.006236141 | Up |
| POLD1 | 5.820663469 | 5.993533625 | 0.011590826 | Up |
| POLD3 | 7.09366 | 7.232160781 | 0.004206159 | Up |
| POLE2 | 4.09571975 | 4.246393594 | 0.005974181 | Up |
| NT5C2 | 9.712373375 | 9.548936125 | 0.000601467 | Down |
| NT5C3B | 7.85347975 | 7.576375625 | 0.000254844 | Down |
| NT5E | 9.017573656 | 8.80687675 | 0.00057262 | Down |
| UPP1 | 6.641213594 | 6.939296438 | 0.000827586 | Up |
| DPYD | 9.428535406 | 9.79395725 | 0.000144687 | Up |
| DPYS | 4.0777355 | 4.146127563 | 0.041251077 | Up |
| ENPP1 | 9.466509531 | 8.895627531 | 7.63E-05 | Down |
| TXNRD2 | 6.919648719 | 7.015654969 | 0.019096774 | Up |
| RRM1 | 8.587227438 | 8.389566594 | 2.41E-05 | Down |
| RRM2 | 5.061020531 | 5.491796844 | 0.001716923 | Up |
| DCTPP1 | 8.369369688 | 8.537904406 | 0.037421803 | Up |
| TYMS | 6.554432688 | 6.7963775 | 0.008725293 | Up |
| TYMP | 8.241860563 | 8.73618375 | 4.34E-05 | Up |
| PNP | 8.22862 | 8.897187 | 7.63E-05 | Up |
| DCK | 7.466716219 | 7.719517094 | 0.000311874 | Up |
| TK2 | 8.46358225 | 8.568721188 | 0.024507787 | Up |
| DTYMK | 5.588833563 | 5.702336969 | 0.010943308 | Up |
| NUDT2 | 6.604575688 | 6.730578531 | 0.013555916 | Up |

# Appendix 3

**Analysis of enrichment**

**Table S3a. Analysis of GO.**

| ONTOLOGY | ID | Description | GeneRatio | pvalue | qvalue | Count |
| --- | --- | --- | --- | --- | --- | --- |
| BP | GO:0072527 | pyrimidine-containing compound metabolic process | 18/41 | 1.59E-32 | 5.62E-30 | 18 |
| BP | GO:0006220 | pyrimidine nucleotide metabolic process | 15/41 | 1.32E-29 | 2.32E-27 | 15 |
| BP | GO:0034404 | nucleobase-containing small molecule biosynthetic process | 17/41 | 8.69E-28 | 1.02E-25 | 17 |
| BP | GO:0009123 | nucleoside monophosphate metabolic process | 15/41 | 1.29E-26 | 1.14E-24 | 15 |
| BP | GO:0009262 | deoxyribonucleotide metabolic process | 12/41 | 1.01E-23 | 5.91E-22 | 12 |
| BP | GO:0072528 | pyrimidine-containing compound biosynthetic process | 12/41 | 1.01E-23 | 5.91E-22 | 12 |
| BP | GO:0006206 | pyrimidine nucleobase metabolic process | 9/41 | 4.77E-21 | 2.40E-19 | 9 |
| BP | GO:0009116 | nucleoside metabolic process | 13/41 | 3.11E-20 | 1.37E-18 | 13 |
| BP | GO:0009141 | nucleoside triphosphate metabolic process | 13/41 | 5.90E-20 | 2.31E-18 | 13 |
| BP | GO:0009165 | nucleotide biosynthetic process | 16/41 | 1.04E-19 | 3.65E-18 | 16 |
| BP | GO:1901293 | nucleoside phosphate biosynthetic process | 16/41 | 1.24E-19 | 3.99E-18 | 16 |
| BP | GO:1901292 | nucleoside phosphate catabolic process | 12/41 | 1.64E-19 | 4.74E-18 | 12 |
| BP | GO:0009394 | 2'-deoxyribonucleotide metabolic process | 10/41 | 1.75E-19 | 4.74E-18 | 10 |
| BP | GO:0019692 | deoxyribose phosphate metabolic process | 10/41 | 3.24E-19 | 8.17E-18 | 10 |
| BP | GO:1901657 | glycosyl compound metabolic process | 13/41 | 5.78E-19 | 1.36E-17 | 13 |
| BP | GO:0009147 | pyrimidine nucleoside triphosphate metabolic process | 9/41 | 8.40E-19 | 1.85E-17 | 9 |
| BP | GO:0009218 | pyrimidine ribonucleotide metabolic process | 9/41 | 4.09E-18 | 8.49E-17 | 9 |
| BP | GO:0006221 | pyrimidine nucleotide biosynthetic process | 9/41 | 5.84E-18 | 1.14E-16 | 9 |
| BP | GO:0009112 | nucleobase metabolic process | 9/41 | 2.13E-17 | 3.95E-16 | 9 |
| BP | GO:0006213 | pyrimidine nucleoside metabolic process | 9/41 | 3.81E-17 | 6.71E-16 | 9 |
| BP | GO:0009219 | pyrimidine deoxyribonucleotide metabolic process | 8/41 | 1.15E-16 | 1.94E-15 | 8 |
| BP | GO:0046434 | organophosphate catabolic process | 12/41 | 3.66E-16 | 5.87E-15 | 12 |
| BP | GO:0015949 | nucleobase-containing small molecule interconversion | 8/41 | 5.19E-16 | 7.96E-15 | 8 |
| BP | GO:0034656 | nucleobase-containing small molecule catabolic process | 9/41 | 9.92E-16 | 1.46E-14 | 9 |
| BP | GO:0009142 | nucleoside triphosphate biosynthetic process | 10/41 | 1.77E-15 | 2.49E-14 | 10 |
| BP | GO:0009162 | deoxyribonucleoside monophosphate metabolic process | 7/41 | 2.56E-15 | 3.47E-14 | 7 |
| BP | GO:0009161 | ribonucleoside monophosphate metabolic process | 9/41 | 2.97E-15 | 3.88E-14 | 9 |
| BP | GO:0009259 | ribonucleotide metabolic process | 15/41 | 5.70E-15 | 7.17E-14 | 15 |
| BP | GO:0009129 | pyrimidine nucleoside monophosphate metabolic process | 7/41 | 6.60E-15 | 7.76E-14 | 7 |
| BP | GO:0009148 | pyrimidine nucleoside triphosphate biosynthetic process | 7/41 | 6.60E-15 | 7.76E-14 | 7 |
| BP | GO:0019693 | ribose phosphate metabolic process | 15/41 | 8.02E-15 | 9.12E-14 | 15 |
| BP | GO:0009163 | nucleoside biosynthetic process | 8/41 | 1.12E-14 | 1.24E-13 | 8 |
| BP | GO:1901659 | glycosyl compound biosynthetic process | 8/41 | 2.70E-14 | 2.88E-13 | 8 |
| BP | GO:0009166 | nucleotide catabolic process | 9/41 | 3.71E-14 | 3.85E-13 | 9 |
| BP | GO:0009263 | deoxyribonucleotide biosynthetic process | 6/41 | 3.55E-13 | 3.58E-12 | 6 |
| BP | GO:0046134 | pyrimidine nucleoside biosynthetic process | 6/41 | 5.67E-13 | 5.55E-12 | 6 |
| BP | GO:0009164 | nucleoside catabolic process | 7/41 | 6.88E-13 | 6.56E-12 | 7 |
| BP | GO:0072529 | pyrimidine-containing compound catabolic process | 7/41 | 2.36E-12 | 2.19E-11 | 7 |
| BP | GO:0006244 | pyrimidine nucleotide catabolic process | 6/41 | 2.73E-12 | 2.47E-11 | 6 |
| BP | GO:0009124 | nucleoside monophosphate biosynthetic process | 7/41 | 4.07E-12 | 3.58E-11 | 7 |
| BP | GO:1901658 | glycosyl compound catabolic process | 7/41 | 4.83E-12 | 4.15E-11 | 7 |
| BP | GO:0009220 | pyrimidine ribonucleotide biosynthetic process | 6/41 | 5.23E-12 | 4.39E-11 | 6 |
| BP | GO:0009176 | pyrimidine deoxyribonucleoside monophosphate metabolic process | 5/41 | 9.42E-12 | 7.73E-11 | 5 |
| BP | GO:0009125 | nucleoside monophosphate catabolic process | 5/41 | 4.79E-11 | 3.84E-10 | 5 |
| BP | GO:0009173 | pyrimidine ribonucleoside monophosphate metabolic process | 5/41 | 7.44E-11 | 5.70E-10 | 5 |
| BP | GO:0046049 | UMP metabolic process | 5/41 | 7.44E-11 | 5.70E-10 | 5 |
| BP | GO:0009200 | deoxyribonucleoside triphosphate metabolic process | 5/41 | 1.62E-10 | 1.21E-09 | 5 |
| BP | GO:0009208 | pyrimidine ribonucleoside triphosphate metabolic process | 5/41 | 3.16E-10 | 2.28E-09 | 5 |
| BP | GO:0009223 | pyrimidine deoxyribonucleotide catabolic process | 5/41 | 3.16E-10 | 2.28E-09 | 5 |
| BP | GO:0009199 | ribonucleoside triphosphate metabolic process | 7/41 | 6.88E-10 | 4.85E-09 | 7 |
| BP | GO:0046135 | pyrimidine nucleoside catabolic process | 5/41 | 1.23E-09 | 8.52E-09 | 5 |
| BP | GO:0009132 | nucleoside diphosphate metabolic process | 8/41 | 1.39E-09 | 9.40E-09 | 8 |
| BP | GO:0009264 | deoxyribonucleotide catabolic process | 5/41 | 2.40E-09 | 1.60E-08 | 5 |
| BP | GO:0072521 | purine-containing compound metabolic process | 11/41 | 2.63E-09 | 1.72E-08 | 11 |
| BP | GO:0032201 | telomere maintenance via semi-conservative replication | 5/41 | 2.94E-09 | 1.85E-08 | 5 |
| BP | GO:0046386 | deoxyribose phosphate catabolic process | 5/41 | 2.94E-09 | 1.85E-08 | 5 |
| BP | GO:0009260 | ribonucleotide biosynthetic process | 8/41 | 6.05E-09 | 3.74E-08 | 8 |
| BP | GO:0008655 | pyrimidine-containing compound salvage | 4/41 | 6.27E-09 | 3.75E-08 | 4 |
| BP | GO:0043097 | pyrimidine nucleoside salvage | 4/41 | 6.27E-09 | 3.75E-08 | 4 |
| BP | GO:0046390 | ribose phosphate biosynthetic process | 8/41 | 8.06E-09 | 4.74E-08 | 8 |
| BP | GO:1901136 | carbohydrate derivative catabolic process | 8/41 | 9.09E-09 | 5.25E-08 | 8 |
| BP | GO:0009265 | 2'-deoxyribonucleotide biosynthetic process | 4/41 | 9.39E-09 | 5.26E-08 | 4 |
| BP | GO:0046385 | deoxyribose phosphate biosynthetic process | 4/41 | 9.39E-09 | 5.26E-08 | 4 |
| BP | GO:0043094 | cellular metabolic compound salvage | 5/41 | 1.00E-08 | 5.49E-08 | 5 |
| BP | GO:0009119 | ribonucleoside metabolic process | 6/41 | 1.01E-08 | 5.49E-08 | 6 |
| BP | GO:0009201 | ribonucleoside triphosphate biosynthetic process | 6/41 | 1.10E-08 | 5.88E-08 | 6 |
| BP | GO:0046940 | nucleoside monophosphate phosphorylation | 4/41 | 1.35E-08 | 7.13E-08 | 4 |
| BP | GO:0032481 | positive regulation of type I interferon production | 6/41 | 1.52E-08 | 7.90E-08 | 6 |
| BP | GO:0009130 | pyrimidine nucleoside monophosphate biosynthetic process | 4/41 | 1.89E-08 | 9.67E-08 | 4 |
| BP | GO:0009261 | ribonucleotide catabolic process | 5/41 | 2.35E-08 | 1.18E-07 | 5 |
| BP | GO:0009209 | pyrimidine ribonucleoside triphosphate biosynthetic process | 4/41 | 2.58E-08 | 1.27E-07 | 4 |
| BP | GO:0006163 | purine nucleotide metabolic process | 10/41 | 2.58E-08 | 1.27E-07 | 10 |
| BP | GO:0009126 | purine nucleoside monophosphate metabolic process | 5/41 | 3.41E-08 | 1.61E-07 | 5 |
| BP | GO:0043174 | nucleoside salvage | 4/41 | 3.43E-08 | 1.61E-07 | 4 |
| BP | GO:0046036 | CTP metabolic process | 4/41 | 3.43E-08 | 1.61E-07 | 4 |
| BP | GO:0046112 | nucleobase biosynthetic process | 4/41 | 5.75E-08 | 2.67E-07 | 4 |
| BP | GO:0072523 | purine-containing compound catabolic process | 5/41 | 8.23E-08 | 3.77E-07 | 5 |
| BP | GO:0046128 | purine ribonucleoside metabolic process | 5/41 | 1.59E-07 | 7.18E-07 | 5 |
| BP | GO:0033260 | nuclear DNA replication | 5/41 | 1.73E-07 | 7.73E-07 | 5 |
| BP | GO:0042278 | purine nucleoside metabolic process | 5/41 | 2.05E-07 | 9.04E-07 | 5 |
| BP | GO:0044786 | cell cycle DNA replication | 5/41 | 2.62E-07 | 1.14E-06 | 5 |
| BP | GO:0044282 | small molecule catabolic process | 9/41 | 2.86E-07 | 1.23E-06 | 9 |
| BP | GO:0032479 | regulation of type I interferon production | 6/41 | 3.21E-07 | 1.36E-06 | 6 |
| BP | GO:0032606 | type I interferon production | 6/41 | 3.36E-07 | 1.41E-06 | 6 |
| BP | GO:0006165 | nucleoside diphosphate phosphorylation | 6/41 | 3.85E-07 | 1.60E-06 | 6 |
| BP | GO:0046939 | nucleotide phosphorylation | 6/41 | 4.03E-07 | 1.65E-06 | 6 |
| BP | GO:0006283 | transcription-coupled nucleotide-excision repair | 5/41 | 5.08E-07 | 2.06E-06 | 5 |
| BP | GO:0009156 | ribonucleoside monophosphate biosynthetic process | 4/41 | 8.50E-07 | 3.41E-06 | 4 |
| BP | GO:0006222 | UMP biosynthetic process | 3/41 | 1.13E-06 | 4.39E-06 | 3 |
| BP | GO:0009133 | nucleoside diphosphate biosynthetic process | 3/41 | 1.13E-06 | 4.39E-06 | 3 |
| BP | GO:0009174 | pyrimidine ribonucleoside monophosphate biosynthetic process | 3/41 | 1.13E-06 | 4.39E-06 | 3 |
| BP | GO:0009167 | purine ribonucleoside monophosphate metabolic process | 4/41 | 1.66E-06 | 6.36E-06 | 4 |
| BP | GO:0009134 | nucleoside diphosphate catabolic process | 3/41 | 2.07E-06 | 7.84E-06 | 3 |
| BP | GO:0006260 | DNA replication | 7/41 | 2.15E-06 | 8.07E-06 | 7 |
| BP | GO:0009150 | purine ribonucleotide metabolic process | 8/41 | 2.29E-06 | 8.49E-06 | 8 |
| BP | GO:0006195 | purine nucleotide catabolic process | 4/41 | 2.93E-06 | 1.08E-05 | 4 |
| BP | GO:0006383 | transcription by RNA polymerase III | 4/41 | 3.20E-06 | 1.16E-05 | 4 |
| BP | GO:0006241 | CTP biosynthetic process | 3/41 | 3.41E-06 | 1.23E-05 | 3 |
| BP | GO:0006289 | nucleotide-excision repair | 5/41 | 3.57E-06 | 1.27E-05 | 5 |
| BP | GO:0046033 | AMP metabolic process | 3/41 | 5.23E-06 | 1.85E-05 | 3 |
| BP | GO:0072522 | purine-containing compound biosynthetic process | 6/41 | 5.45E-06 | 1.90E-05 | 6 |
| BP | GO:0006541 | glutamine metabolic process | 3/41 | 1.64E-05 | 5.66E-05 | 3 |
| BP | GO:0042454 | ribonucleoside catabolic process | 3/41 | 1.87E-05 | 6.40E-05 | 3 |
| BP | GO:0006353 | DNA-templated transcription, termination | 4/41 | 2.09E-05 | 7.08E-05 | 4 |
| BP | GO:0006261 | DNA-dependent DNA replication | 5/41 | 2.21E-05 | 7.41E-05 | 5 |
| BP | GO:0050434 | positive regulation of viral transcription | 3/41 | 2.39E-05 | 7.96E-05 | 3 |
| BP | GO:0000723 | telomere maintenance | 5/41 | 2.49E-05 | 8.20E-05 | 5 |
| BP | GO:0032200 | telomere organization | 5/41 | 3.61E-05 | 0.00011793 | 5 |
| BP | GO:0006362 | transcription elongation from RNA polymerase I promoter | 3/41 | 3.72E-05 | 0.000120203 | 3 |
| BP | GO:0006363 | termination of RNA polymerase I transcription | 3/41 | 4.11E-05 | 0.000131673 | 3 |
| BP | GO:0006370 | 7-methylguanosine mRNA capping | 3/41 | 4.97E-05 | 0.000157906 | 3 |
| BP | GO:0009452 | 7-methylguanosine RNA capping | 3/41 | 5.44E-05 | 0.000169866 | 3 |
| BP | GO:0036260 | RNA capping | 3/41 | 5.44E-05 | 0.000169866 | 3 |
| BP | GO:0071897 | DNA biosynthetic process | 5/41 | 6.06E-05 | 0.000187584 | 5 |
| BP | GO:0006361 | transcription initiation from RNA polymerase I promoter | 3/41 | 7.63E-05 | 0.000233889 | 3 |
| BP | GO:0006270 | DNA replication initiation | 3/41 | 8.91E-05 | 0.000270744 | 3 |
| BP | GO:0098781 | ncRNA transcription | 4/41 | 9.06E-05 | 0.000273146 | 4 |
| BP | GO:0019985 | translesion synthesis | 3/41 | 0.000103174 | 0.000305734 | 3 |
| BP | GO:0046782 | regulation of viral transcription | 3/41 | 0.000103174 | 0.000305734 | 3 |
| BP | GO:0006354 | DNA-templated transcription, elongation | 4/41 | 0.000127347 | 0.00037422 | 4 |
| BP | GO:0006301 | postreplication repair | 3/41 | 0.000195645 | 0.00057017 | 3 |
| BP | GO:0006152 | purine nucleoside catabolic process | 2/41 | 0.00020517 | 0.000583462 | 2 |
| BP | GO:0006228 | UTP biosynthetic process | 2/41 | 0.00020517 | 0.000583462 | 2 |
| BP | GO:0046130 | purine ribonucleoside catabolic process | 2/41 | 0.00020517 | 0.000583462 | 2 |
| BP | GO:0000731 | DNA synthesis involved in DNA repair | 3/41 | 0.000207071 | 0.000584159 | 3 |
| BP | GO:0009185 | ribonucleoside diphosphate metabolic process | 4/41 | 0.000225145 | 0.000630105 | 4 |
| BP | GO:0045815 | positive regulation of gene expression, epigenetic | 3/41 | 0.0002707 | 0.000751631 | 3 |
| BP | GO:0046051 | UTP metabolic process | 2/41 | 0.000300087 | 0.000826721 | 2 |
| BP | GO:0000082 | G1/S transition of mitotic cell cycle | 5/41 | 0.000309842 | 0.000846978 | 5 |
| BP | GO:0001819 | positive regulation of cytokine production | 6/41 | 0.000337945 | 0.000916693 | 6 |
| BP | GO:0009143 | nucleoside triphosphate catabolic process | 2/41 | 0.000412618 | 0.001099279 | 2 |
| BP | GO:0009151 | purine deoxyribonucleotide metabolic process | 2/41 | 0.000412618 | 0.001099279 | 2 |
| BP | GO:0009206 | purine ribonucleoside triphosphate biosynthetic process | 3/41 | 0.000414609 | 0.001099279 | 3 |
| BP | GO:0006360 | transcription by RNA polymerase I | 3/41 | 0.000433091 | 0.001131272 | 3 |
| BP | GO:0009145 | purine nucleoside triphosphate biosynthetic process | 3/41 | 0.000433091 | 0.001131272 | 3 |
| BP | GO:0044843 | cell cycle G1/S phase transition | 5/41 | 0.000447611 | 0.001160601 | 5 |
| BP | GO:0043101 | purine-containing compound salvage | 2/41 | 0.000475443 | 0.001223768 | 2 |
| BP | GO:0035019 | somatic stem cell population maintenance | 3/41 | 0.000491694 | 0.001256426 | 3 |
| BP | GO:0042795 | snRNA transcription by RNA polymerase II | 3/41 | 0.000555155 | 0.001408383 | 3 |
| BP | GO:0009064 | glutamine family amino acid metabolic process | 3/41 | 0.000577416 | 0.001444079 | 3 |
| BP | GO:0009301 | snRNA transcription | 3/41 | 0.000577416 | 0.001444079 | 3 |
| BP | GO:0009205 | purine ribonucleoside triphosphate metabolic process | 3/41 | 0.000697293 | 0.001731603 | 3 |
| BP | GO:0006520 | cellular amino acid metabolic process | 5/41 | 0.000720497 | 0.001776714 | 5 |
| BP | GO:0009144 | purine nucleoside triphosphate metabolic process | 3/41 | 0.000860753 | 0.002100919 | 3 |
| BP | GO:0006164 | purine nucleotide biosynthetic process | 4/41 | 0.000863885 | 0.002100919 | 4 |
| BP | GO:0006368 | transcription elongation from RNA polymerase II promoter | 3/41 | 0.000982163 | 0.002372202 | 3 |
| BP | GO:0048524 | positive regulation of viral process | 3/41 | 0.001014119 | 0.002432723 | 3 |
| BP | GO:0042451 | purine nucleoside biosynthetic process | 2/41 | 0.001035944 | 0.002435376 | 2 |
| BP | GO:0042455 | ribonucleoside biosynthetic process | 2/41 | 0.001035944 | 0.002435376 | 2 |
| BP | GO:0046129 | purine ribonucleoside biosynthetic process | 2/41 | 0.001035944 | 0.002435376 | 2 |
| BP | GO:0006297 | nucleotide-excision repair, DNA gap filling | 2/41 | 0.001133045 | 0.002611421 | 2 |
| BP | GO:0006359 | regulation of transcription by RNA polymerase III | 2/41 | 0.001133045 | 0.002611421 | 2 |
| BP | GO:0009127 | purine nucleoside monophosphate biosynthetic process | 2/41 | 0.001133045 | 0.002611421 | 2 |
| BP | GO:0008543 | fibroblast growth factor receptor signaling pathway | 3/41 | 0.001890913 | 0.004329841 | 3 |
| BP | GO:0006352 | DNA-templated transcription, initiation | 4/41 | 0.002045972 | 0.004654673 | 4 |
| BP | GO:0060964 | regulation of gene silencing by miRNA | 3/41 | 0.002297988 | 0.005194508 | 3 |
| BP | GO:0032728 | positive regulation of interferon-beta production | 2/41 | 0.002332329 | 0.005238553 | 2 |
| BP | GO:0051607 | defense response to virus | 4/41 | 0.00239324 | 0.005307748 | 4 |
| BP | GO:0140546 | defense response to symbiont | 4/41 | 0.00239324 | 0.005307748 | 4 |
| BP | GO:0060147 | regulation of posttranscriptional gene silencing | 3/41 | 0.00246357 | 0.005429578 | 3 |
| BP | GO:0060966 | regulation of gene silencing by RNA | 3/41 | 0.002520363 | 0.005520246 | 3 |
| BP | GO:0000083 | regulation of transcription involved in G1/S transition of mitotic cell cycle | 2/41 | 0.002621076 | 0.005670394 | 2 |
| BP | GO:0009154 | purine ribonucleotide catabolic process | 2/41 | 0.002621076 | 0.005670394 | 2 |
| BP | GO:0006296 | nucleotide-excision repair, DNA incision, 5'-to lesion | 2/41 | 0.002925796 | 0.006291024 | 2 |
| BP | GO:0006298 | mismatch repair | 2/41 | 0.003084103 | 0.006591224 | 2 |
| BP | GO:0009135 | purine nucleoside diphosphate metabolic process | 3/41 | 0.003133284 | 0.006616138 | 3 |
| BP | GO:0009179 | purine ribonucleoside diphosphate metabolic process | 3/41 | 0.003133284 | 0.006616138 | 3 |
| BP | GO:0060249 | anatomical structure homeostasis | 5/41 | 0.003243934 | 0.006733917 | 5 |
| BP | GO:0033683 | nucleotide-excision repair, DNA incision | 2/41 | 0.003246351 | 0.006733917 | 2 |
| BP | GO:0042769 | DNA damage response, detection of DNA damage | 2/41 | 0.003246351 | 0.006733917 | 2 |
| BP | GO:1901068 | guanosine-containing compound metabolic process | 2/41 | 0.003412524 | 0.007037215 | 2 |
| BP | GO:0044344 | cellular response to fibroblast growth factor stimulus | 3/41 | 0.003612318 | 0.007405915 | 3 |
| BP | GO:0060968 | regulation of gene silencing | 3/41 | 0.003756947 | 0.007657909 | 3 |
| BP | GO:0019827 | stem cell population maintenance | 3/41 | 0.003905076 | 0.007914099 | 3 |
| BP | GO:0071774 | response to fibroblast growth factor | 3/41 | 0.004056731 | 0.00812802 | 3 |
| BP | GO:0098727 | maintenance of cell number | 3/41 | 0.004056731 | 0.00812802 | 3 |
| BP | GO:0090305 | nucleic acid phosphodiester bond hydrolysis | 4/41 | 0.004239421 | 0.008446066 | 4 |
| BP | GO:0046034 | ATP metabolic process | 4/41 | 0.004646127 | 0.009204331 | 4 |
| BP | GO:0032648 | regulation of interferon-beta production | 2/41 | 0.005286403 | 0.01041426 | 2 |
| BP | GO:0032608 | interferon-beta production | 2/41 | 0.005706598 | 0.011179592 | 2 |
| BP | GO:0009152 | purine ribonucleotide biosynthetic process | 3/41 | 0.006461294 | 0.012588155 | 3 |
| BP | GO:0006754 | ATP biosynthetic process | 2/41 | 0.006591353 | 0.012770986 | 2 |
| BP | GO:0019083 | viral transcription | 3/41 | 0.006982061 | 0.013454071 | 3 |
| BP | GO:0009615 | response to virus | 4/41 | 0.007503531 | 0.014380336 | 4 |
| BP | GO:0050792 | regulation of viral process | 3/41 | 0.00763914 | 0.014561091 | 3 |
| BP | GO:0006367 | transcription initiation from RNA polymerase II promoter | 3/41 | 0.007752094 | 0.014696953 | 3 |
| BP | GO:1901605 | alpha-amino acid metabolic process | 3/41 | 0.00821382 | 0.01548905 | 3 |
| BP | GO:0043903 | regulation of biological process involved in symbiotic interaction | 3/41 | 0.00893635 | 0.016761911 | 3 |
| BP | GO:0019080 | viral gene expression | 3/41 | 0.009060289 | 0.016904466 | 3 |
| BP | GO:0040029 | regulation of gene expression, epigenetic | 3/41 | 0.009956231 | 0.018478324 | 3 |
| BP | GO:0031100 | animal organ regeneration | 2/41 | 0.010989542 | 0.020289317 | 2 |
| BP | GO:0034644 | cellular response to UV | 2/41 | 0.015683196 | 0.028804115 | 2 |
| BP | GO:0009991 | response to extracellular stimulus | 4/41 | 0.019553278 | 0.035725925 | 4 |
| BP | GO:0016311 | dephosphorylation | 4/41 | 0.021488901 | 0.038736435 | 4 |
| BP | GO:0035999 | tetrahydrofolate interconversion | 1/41 | 0.02153052 | 0.038736435 | 1 |
| BP | GO:0051593 | response to folic acid | 1/41 | 0.02153052 | 0.038736435 | 1 |
| BP | GO:0006183 | GTP biosynthetic process | 1/41 | 0.02365853 | 0.042135075 | 1 |
| BP | GO:0046710 | GDP metabolic process | 1/41 | 0.02365853 | 0.042135075 | 1 |
| BP | GO:0031953 | negative regulation of protein autophosphorylation | 1/41 | 0.025782025 | 0.045642472 | 1 |
| BP | GO:0006757 | ATP generation from ADP | 2/41 | 0.025886775 | 0.045642472 | 2 |
| BP | GO:0055062 | phosphate ion homeostasis | 1/41 | 0.027901014 | 0.047994041 | 1 |
| BP | GO:0060576 | intestinal epithelial cell development | 1/41 | 0.027901014 | 0.047994041 | 1 |
| BP | GO:0070914 | UV-damage excision repair | 1/41 | 0.027901014 | 0.047994041 | 1 |
| BP | GO:0072506 | trivalent inorganic anion homeostasis | 1/41 | 0.027901014 | 0.047994041 | 1 |
| BP | GO:1901070 | guanosine-containing compound biosynthetic process | 1/41 | 0.027901014 | 0.047994041 | 1 |
| BP | GO:0046031 | ADP metabolic process | 2/41 | 0.0288731 | 0.049425082 | 2 |
| BP | GO:0030002 | cellular anion homeostasis | 1/41 | 0.030015505 | 0.049459884 | 1 |
| BP | GO:0043173 | nucleotide salvage | 1/41 | 0.030015505 | 0.049459884 | 1 |
| BP | GO:0046040 | IMP metabolic process | 1/41 | 0.030015505 | 0.049459884 | 1 |
| BP | GO:0046131 | pyrimidine ribonucleoside metabolic process | 1/41 | 0.030015505 | 0.049459884 | 1 |
| BP | GO:0046133 | pyrimidine ribonucleoside catabolic process | 1/41 | 0.030015505 | 0.049459884 | 1 |
| BP | GO:0046325 | negative regulation of glucose import | 1/41 | 0.030015505 | 0.049459884 | 1 |
| BP | GO:0046415 | urate metabolic process | 1/41 | 0.030015505 | 0.049459884 | 1 |
| BP | GO:0051290 | protein heterotetramerization | 1/41 | 0.030015505 | 0.049459884 | 1 |
| BP | GO:0002070 | epithelial cell maturation | 1/41 | 0.03212551 | 0.052446617 | 1 |
| BP | GO:0051969 | regulation of transmission of nerve impulse | 1/41 | 0.03212551 | 0.052446617 | 1 |
| BP | GO:0071482 | cellular response to light stimulus | 2/41 | 0.034295681 | 0.055731521 | 2 |
| BP | GO:0051384 | response to glucocorticoid | 2/41 | 0.034764288 | 0.05623388 | 2 |
| BP | GO:0001889 | liver development | 2/41 | 0.036185144 | 0.057452213 | 2 |
| BP | GO:0030730 | sequestering of triglyceride | 1/41 | 0.036332094 | 0.057452213 | 1 |
| BP | GO:0031000 | response to caffeine | 1/41 | 0.036332094 | 0.057452213 | 1 |
| BP | GO:0042790 | nucleolar large rRNA transcription by RNA polymerase I | 1/41 | 0.036332094 | 0.057452213 | 1 |
| BP | GO:0055089 | fatty acid homeostasis | 1/41 | 0.036332094 | 0.057452213 | 1 |
| BP | GO:0061008 | hepaticobiliary system development | 2/41 | 0.037144783 | 0.058475105 | 2 |
| BP | GO:0032026 | response to magnesium ion | 1/41 | 0.038428691 | 0.05996093 | 1 |
| BP | GO:0033189 | response to vitamin A | 1/41 | 0.038428691 | 0.05996093 | 1 |
| BP | GO:0009411 | response to UV | 2/41 | 0.040082145 | 0.061856828 | 2 |
| BP | GO:0006144 | purine nucleobase metabolic process | 1/41 | 0.040520839 | 0.061856828 | 1 |
| BP | GO:0006271 | DNA strand elongation involved in DNA replication | 1/41 | 0.040520839 | 0.061856828 | 1 |
| BP | GO:0010829 | negative regulation of glucose transmembrane transport | 1/41 | 0.040520839 | 0.061856828 | 1 |
| BP | GO:0046653 | tetrahydrofolate metabolic process | 1/41 | 0.040520839 | 0.061856828 | 1 |
| BP | GO:0031960 | response to corticosteroid | 2/41 | 0.043105117 | 0.065518213 | 2 |
| BP | GO:0000002 | mitochondrial genome maintenance | 1/41 | 0.044691819 | 0.066778587 | 1 |
| BP | GO:0009168 | purine ribonucleoside monophosphate biosynthetic process | 1/41 | 0.044691819 | 0.066778587 | 1 |
| BP | GO:0030502 | negative regulation of bone mineralization | 1/41 | 0.044691819 | 0.066778587 | 1 |
| BP | GO:0042276 | error-prone translesion synthesis | 1/41 | 0.044691819 | 0.066778587 | 1 |
| BP | GO:0060575 | intestinal epithelial cell differentiation | 1/41 | 0.04677067 | 0.069297543 | 1 |
| BP | GO:0070987 | error-free translesion synthesis | 1/41 | 0.04677067 | 0.069297543 | 1 |
| BP | GO:0043467 | regulation of generation of precursor metabolites and energy | 2/41 | 0.047264439 | 0.069736125 | 2 |
| CC | GO:0061695 | transferase complex, transferring phosphorus-containing groups | 13/41 | 2.73E-15 | 1.09E-13 | 13 |
| CC | GO:0055029 | nuclear DNA-directed RNA polymerase complex | 10/41 | 1.05E-14 | 1.54E-13 | 10 |
| CC | GO:0000428 | DNA-directed RNA polymerase complex | 10/41 | 1.16E-14 | 1.54E-13 | 10 |
| CC | GO:0030880 | RNA polymerase complex | 10/41 | 1.71E-14 | 1.71E-13 | 10 |
| CC | GO:0005666 | RNA polymerase III complex | 6/41 | 1.07E-12 | 8.54E-12 | 6 |
| CC | GO:0042575 | DNA polymerase complex | 5/41 | 4.81E-10 | 3.21E-09 | 5 |
| CC | GO:0043601 | nuclear replisome | 4/41 | 1.19E-07 | 6.81E-07 | 4 |
| CC | GO:0030894 | replisome | 4/41 | 1.73E-07 | 8.63E-07 | 4 |
| CC | GO:0043596 | nuclear replication fork | 4/41 | 8.37E-07 | 3.72E-06 | 4 |
| CC | GO:0005736 | RNA polymerase I complex | 3/41 | 2.42E-06 | 9.70E-06 | 3 |
| CC | GO:0005665 | RNA polymerase II, core complex | 3/41 | 3.85E-06 | 1.40E-05 | 3 |
| CC | GO:0005657 | replication fork | 4/41 | 1.03E-05 | 3.45E-05 | 4 |
| CC | GO:0000228 | nuclear chromosome | 5/41 | 0.000170254 | 0.000523859 | 5 |
| CC | GO:0016591 | RNA polymerase II, holoenzyme | 3/41 | 0.000608791 | 0.001739402 | 3 |
| CC | GO:0032993 | protein-DNA complex | 4/41 | 0.000931503 | 0.002484008 | 4 |
| CC | GO:0005759 | mitochondrial matrix | 5/41 | 0.003068817 | 0.007672042 | 5 |
| CC | GO:0000109 | nucleotide-excision repair complex | 1/41 | 0.024922716 | 0.058641684 | 1 |
| CC | GO:0001650 | fibrillar center | 2/41 | 0.03444844 | 0.076552089 | 2 |
| MF | GO:0016779 | nucleotidyltransferase activity | 12/41 | 7.00E-17 | 2.49E-15 | 12 |
| MF | GO:0003899 | DNA-directed 5'-3' RNA polymerase activity | 9/41 | 1.10E-16 | 2.49E-15 | 9 |
| MF | GO:0034062 | 5'-3' RNA polymerase activity | 9/41 | 2.90E-16 | 3.29E-15 | 9 |
| MF | GO:0097747 | RNA polymerase activity | 9/41 | 2.90E-16 | 3.29E-15 | 9 |
| MF | GO:0019205 | nucleobase-containing compound kinase activity | 7/41 | 4.15E-12 | 3.75E-11 | 7 |
| MF | GO:0004550 | nucleoside diphosphate kinase activity | 5/41 | 3.64E-10 | 2.75E-09 | 5 |
| MF | GO:0016776 | phosphotransferase activity, phosphate group as acceptor | 5/41 | 2.36E-08 | 1.53E-07 | 5 |
| MF | GO:0050145 | nucleoside monophosphate kinase activity | 4/41 | 1.02E-07 | 5.74E-07 | 4 |
| MF | GO:0140098 | catalytic activity, acting on RNA | 9/41 | 1.43E-07 | 7.18E-07 | 9 |
| MF | GO:0008253 | 5'-nucleotidase activity | 3/41 | 2.92E-06 | 1.32E-05 | 3 |
| MF | GO:0017110 | nucleoside-diphosphatase activity | 3/41 | 3.71E-06 | 1.53E-05 | 3 |
| MF | GO:0008252 | nucleotidase activity | 3/41 | 4.63E-06 | 1.75E-05 | 3 |
| MF | GO:0003887 | DNA-directed DNA polymerase activity | 3/41 | 2.31E-05 | 8.03E-05 | 3 |
| MF | GO:0034061 | DNA polymerase activity | 3/41 | 7.04E-05 | 0.000227528 | 3 |
| MF | GO:0016879 | ligase activity, forming carbon-nitrogen bonds | 3/41 | 0.000157117 | 0.000474107 | 3 |
| MF | GO:0016763 | pentosyltransferase activity | 3/41 | 0.000189034 | 0.000534768 | 3 |
| MF | GO:0047429 | nucleoside-triphosphate diphosphatase activity | 2/41 | 0.000264868 | 0.000705222 | 2 |
| MF | GO:0004551 | nucleotide diphosphatase activity | 2/41 | 0.000903409 | 0.00227173 | 2 |
| MF | GO:0042578 | phosphoric ester hydrolase activity | 5/41 | 0.001295395 | 0.003085982 | 5 |
| MF | GO:0016791 | phosphatase activity | 4/41 | 0.003282736 | 0.007429349 | 4 |
| MF | GO:0051539 | 4 iron, 4 sulfur cluster binding | 2/41 | 0.003968522 | 0.008553706 | 2 |
| MF | GO:0016874 | ligase activity | 3/41 | 0.0057387 | 0.011806895 | 3 |
| MF | GO:0016597 | amino acid binding | 2/41 | 0.006485096 | 0.012762431 | 2 |
| MF | GO:0031406 | carboxylic acid binding | 3/41 | 0.008009284 | 0.015105228 | 3 |
| MF | GO:0051536 | iron-sulfur cluster binding | 2/41 | 0.009838261 | 0.017127337 | 2 |
| MF | GO:0051540 | metal cluster binding | 2/41 | 0.009838261 | 0.017127337 | 2 |
| MF | GO:0140097 | catalytic activity, acting on DNA | 3/41 | 0.01060198 | 0.017773299 | 3 |
| MF | GO:0050660 | flavin adenine dinucleotide binding | 2/41 | 0.0141362 | 0.022583639 | 2 |
| MF | GO:0004527 | exonuclease activity | 2/41 | 0.014469285 | 0.022583639 | 2 |
| MF | GO:0016884 | carbon-nitrogen ligase activity, with glutamine as amido-N-donor | 1/41 | 0.022140911 | 0.033405584 | 1 |
| MF | GO:0016757 | glycosyltransferase activity | 3/41 | 0.022946886 | 0.033504791 | 3 |
| MF | GO:0008296 | 3'-5'-exodeoxyribonuclease activity | 1/41 | 0.024328515 | 0.033737849 | 1 |
| MF | GO:0005542 | folic acid binding | 1/41 | 0.026511344 | 0.033737849 | 1 |
| MF | GO:0016668 | oxidoreductase activity, acting on a sulfur group of donors, NAD(P) as acceptor | 1/41 | 0.026511344 | 0.033737849 | 1 |
| MF | GO:0042301 | phosphate ion binding | 1/41 | 0.026511344 | 0.033737849 | 1 |
| MF | GO:0043177 | organic acid binding | 2/41 | 0.026833359 | 0.033737849 | 2 |
| MF | GO:0016810 | hydrolase activity, acting on carbon-nitrogen (but not peptide) bonds | 2/41 | 0.028152453 | 0.03417298 | 2 |
| MF | GO:1990825 | sequence-specific mRNA binding | 1/41 | 0.028689409 | 0.03417298 | 1 |
| MF | GO:0000900 | translation repressor activity, mRNA regulatory element binding | 1/41 | 0.033031285 | 0.038335904 | 1 |
| MF | GO:0004529 | exodeoxyribonuclease activity | 1/41 | 0.048079246 | 0.051814726 | 1 |
| MF | GO:0005158 | insulin receptor binding | 1/41 | 0.048079246 | 0.051814726 | 1 |
| MF | GO:0016895 | exodeoxyribonuclease activity, producing 5'-phosphomonoesters | 1/41 | 0.048079246 | 0.051814726 | 1 |

**Table S3b. Analysis of KEGG.**

| ID | Description | BgRatio | pvalue | qvalue | Count |
| --- | --- | --- | --- | --- | --- |
| hsa00240 | Pyrimidine metabolism | 99/5894 | 3.53E-66 | 3.35E-65 | 36 |
| hsa00230 | Purine metabolism | 162/5894 | 1.60E-29 | 7.56E-29 | 24 |
| hsa03020 | RNA polymerase | 29/5894 | 2.98E-10 | 9.41E-10 | 7 |
| hsa03030 | DNA replication | 36/5894 | 2.41E-06 | 5.71E-06 | 5 |
| hsa00760 | Nicotinate and nicotinamide metabolism | 24/5894 | 1.28E-05 | 2.42E-05 | 4 |
| hsa00983 | Drug metabolism - other enzymes | 52/5894 | 1.55E-05 | 2.44E-05 | 5 |
| hsa00770 | Pantothenate and CoA biosynthesis | 16/5894 | 0.000120582 | 0.000163194 | 3 |
| hsa03410 | Base excision repair | 34/5894 | 0.001192038 | 0.001411624 | 3 |
| hsa03420 | Nucleotide excision repair | 45/5894 | 0.002695742 | 0.002837623 | 3 |
| hsa04623 | Cytosolic DNA-sensing pathway | 56/5894 | 0.005022569 | 0.004758223 | 3 |
| hsa00410 | beta-Alanine metabolism | 22/5894 | 0.008185145 | 0.007049407 | 2 |
| hsa03430 | Mismatch repair | 23/5894 | 0.008929448 | 0.007049565 | 2 |
| hsa03440 | Homologous recombination | 28/5894 | 0.013081403 | 0.009533007 | 2 |
| hsa00480 | Glutathione metabolism | 50/5894 | 0.038896974 | 0.026321261 | 2 |

# Appendix 4

**LASSO and** **SVM-RFE genes**

**Table S4a. LASSO genes.**

| CAD | POLA2 | DCK | ENTPD5 |
| --- | --- | --- | --- |
| CMPK1 | NT5C2 | DTYMK | PNP |
| CMPK2 | RRM1 |  |  |

**Table S4b. SVM-RFE genes.**

| CMPK1 | POLD1 | RRM2 | RRM1 |
| --- | --- | --- | --- |
| NT5C2 | TK2 | POLR2K | CMPK2 |
| CMPK1 | POLD1 | RRM2 | RRM1 |
| NT5C2 | TK2 | POLR2K | CMPK2 |

**.**

**Table S4c. InterGenes.**

| CMPK1 | NT5C2 | RRM1 | CMPK2 |
| --- | --- | --- | --- |

# Appendix 5

**GSEA analysis**

**Table 5a. NT5C2 of GSEA analysis.**

| ID | setSize | NES | pvalue | qvalue | rank |
| --- | --- | --- | --- | --- | --- |
| KEGG_B_CELL_RECEPTOR_SIGNALING_PATHWAY | 75 | -2.377796207 | 9.42E-09 | 1.03E-06 | 3546 |
| KEGG_CHEMOKINE_SIGNALING_PATHWAY | 186 | -2.05228506 | 1.67E-08 | 1.03E-06 | 3588 |
| KEGG_CYTOKINE_CYTOKINE_RECEPTOR_INTERACTION | 256 | -1.899511813 | 7.51E-08 | 3.08E-06 | 3864 |
| KEGG_FC_GAMMA_R_MEDIATED_PHAGOCYTOSIS | 94 | -2.169241611 | 1.27E-07 | 3.92E-06 | 4626 |
| KEGG_HEMATOPOIETIC_CELL_LINEAGE | 85 | -2.177317212 | 8.60E-07 | 2.12E-05 | 2207 |
| KEGG_LEUKOCYTE_TRANSENDOTHELIAL_MIGRATION | 113 | -1.943182588 | 2.91E-06 | 5.97E-05 | 3527 |
| KEGG_TASTE_TRANSDUCTION | 50 | 2.169058423 | 5.27E-06 | 9.27E-05 | 1189 |
| KEGG_VEGF_SIGNALING_PATHWAY | 74 | -2.107813567 | 6.45E-06 | 9.93E-05 | 4213 |
| KEGG_SYSTEMIC_LUPUS_ERYTHEMATOSUS | 53 | -2.163809157 | 8.40E-06 | 0.000104218 | 2021 |
| KEGG_ACUTE_MYELOID_LEUKEMIA | 57 | -2.138527791 | 8.46E-06 | 0.000104218 | 5813 |
| KEGG_GLYCOSYLPHOSPHATIDYLINOSITOL_GPI_ANCHOR_BIOSYNTHESIS | 25 | 2.144558756 | 1.34E-05 | 0.00014988 | 3454 |
| KEGG_T_CELL_RECEPTOR_SIGNALING_PATHWAY | 107 | -1.915248987 | 2.34E-05 | 0.00022125 | 4213 |
| KEGG_ALZHEIMERS_DISEASE | 145 | -1.828605275 | 2.17E-05 | 0.00022125 | 6887 |
| KEGG_NOTCH_SIGNALING_PATHWAY | 47 | -2.064125887 | 3.85E-05 | 0.000338729 | 5293 |
| KEGG_LYSOSOME | 120 | -1.865385884 | 4.24E-05 | 0.00034799 | 6806 |
| KEGG_FC_EPSILON_RI_SIGNALING_PATHWAY | 77 | -1.949200649 | 5.48E-05 | 0.000421698 | 3329 |
| KEGG_NOD_LIKE_RECEPTOR_SIGNALING_PATHWAY | 62 | -1.909758884 | 8.16E-05 | 0.000590895 | 3546 |
| KEGG_LEISHMANIA_INFECTION | 69 | -1.937944005 | 9.44E-05 | 0.00061219 | 2652 |
| KEGG_COMPLEMENT_AND_COAGULATION_CASCADES | 68 | -1.927769365 | 9.17E-05 | 0.00061219 | 3695 |
| KEGG_NATURAL_KILLER_CELL_MEDIATED_CYTOTOXICITY | 128 | -1.713365339 | 0.000105933 | 0.000652324 | 3118 |
| KEGG_FOCAL_ADHESION | 196 | -1.570432573 | 0.000275482 | 0.001615609 | 2542 |
| KEGG_ECM_RECEPTOR_INTERACTION | 83 | -1.790416462 | 0.000472821 | 0.002646895 | 2375 |
| KEGG_PRION_DISEASES | 35 | -1.841644357 | 0.001021817 | 0.005037728 | 2697 |
| KEGG_NON_SMALL_CELL_LUNG_CANCER | 54 | -1.755133267 | 0.001022616 | 0.005037728 | 3427 |
| KEGG_CELL_ADHESION_MOLECULES_CAMS | 131 | -1.583535254 | 0.000957026 | 0.005037728 | 3527 |
| KEGG_PATHWAYS_IN_CANCER | 322 | -1.374040868 | 0.001426803 | 0.006758541 | 3427 |
| KEGG_OXIDATIVE_PHOSPHORYLATION | 101 | -1.637877502 | 0.001679304 | 0.007659982 | 7020 |
| KEGG_PRIMARY_IMMUNODEFICIENCY | 35 | -1.762287992 | 0.002235269 | 0.009831821 | 3438 |
| KEGG_BLADDER_CANCER | 41 | -1.758342453 | 0.002723743 | 0.011200157 | 4213 |
| KEGG_CHRONIC_MYELOID_LEUKEMIA | 73 | -1.647203196 | 0.002728243 | 0.011200157 | 5813 |
| KEGG_TOLL_LIKE_RECEPTOR_SIGNALING_PATHWAY | 99 | -1.560459912 | 0.002930756 | 0.011643412 | 4797 |
| KEGG_GLIOMA | 65 | -1.578201905 | 0.003362733 | 0.012942096 | 5813 |
| KEGG_ALDOSTERONE_REGULATED_SODIUM_REABSORPTION | 42 | -1.756324311 | 0.003848592 | 0.014363166 | 4811 |
| KEGG_PROPANOATE_METABOLISM | 31 | 1.751684014 | 0.004062547 | 0.014715728 | 5384 |
| KEGG_PPAR_SIGNALING_PATHWAY | 69 | -1.659647019 | 0.004527221 | 0.015930372 | 4520 |
| KEGG_MATURITY_ONSET_DIABETES_OF_THE_YOUNG | 25 | -1.774954738 | 0.004685214 | 0.016028364 | 5452 |
| KEGG_TYPE_II_DIABETES_MELLITUS | 47 | -1.623376127 | 0.005764467 | 0.019187558 | 5507 |
| KEGG_JAK_STAT_SIGNALING_PATHWAY | 152 | -1.457529023 | 0.005970855 | 0.019351526 | 2945 |
| KEGG_GNRH_SIGNALING_PATHWAY | 99 | -1.491443847 | 0.008803458 | 0.026444276 | 3324 |
| KEGG_MAPK_SIGNALING_PATHWAY | 264 | -1.369044324 | 0.008595339 | 0.026444276 | 3709 |
| KEGG_ENDOCYTOSIS | 180 | -1.368464615 | 0.008507958 | 0.026444276 | 3318 |
| KEGG_DRUG_METABOLISM_OTHER_ENZYMES | 41 | -1.62273185 | 0.009377783 | 0.027498762 | 4602 |
| KEGG_VIBRIO_CHOLERAE_INFECTION | 54 | -1.525964655 | 0.010619377 | 0.030415352 | 3003 |
| KEGG_VALINE_LEUCINE_AND_ISOLEUCINE_DEGRADATION | 43 | 1.618030202 | 0.010903479 | 0.030519307 | 5156 |
| KEGG_REGULATION_OF_ACTIN_CYTOSKELETON | 210 | -1.323439081 | 0.0117189 | 0.032072779 | 3488 |
| KEGG_APOPTOSIS | 86 | -1.484729285 | 0.012324793 | 0.03299773 | 3688 |
| KEGG_BUTANOATE_METABOLISM | 33 | 1.575346754 | 0.017876853 | 0.044033512 | 2216 |
| KEGG_EPITHELIAL_CELL_SIGNALING_IN_HELICOBACTER_PYLORI_INFECTION | 67 | -1.446536504 | 0.017748494 | 0.044033512 | 3546 |
| KEGG_RIBOSOME | 86 | -1.444037095 | 0.017205762 | 0.044033512 | 9015 |
| KEGG_OLFACTORY_TRANSDUCTION | 372 | -1.210706277 | 0.017437513 | 0.044033512 | 5066 |
| KEGG_GLUTATHIONE_METABOLISM | 49 | -1.47495035 | 0.01881463 | 0.045434711 | 4084 |
| KEGG_RENIN_ANGIOTENSIN_SYSTEM | 17 | -1.635810507 | 0.019193167 | 0.0454575 | 1362 |
| KEGG_PROSTATE_CANCER | 89 | -1.365599387 | 0.019569496 | 0.0454743 | 6392 |
| KEGG_PHOSPHATIDYLINOSITOL_SIGNALING_SYSTEM | 76 | -1.381697101 | 0.019979003 | 0.045566148 | 3329 |
| KEGG_INTESTINAL_IMMUNE_NETWORK_FOR_IGA_PRODUCTION | 47 | -1.44794732 | 0.027819636 | 0.062294688 | 4797 |
| KEGG_THYROID_CANCER | 29 | -1.523885705 | 0.03128469 | 0.068802795 | 4213 |
| KEGG_ARACHIDONIC_ACID_METABOLISM | 55 | -1.40392022 | 0.03637429 | 0.077237603 | 6638 |
| KEGG_NEUROACTIVE_LIGAND_RECEPTOR_INTERACTION | 270 | -1.248694801 | 0.035896709 | 0.077237603 | 5286 |
| KEGG_MELANOGENESIS | 101 | -1.334667819 | 0.03875969 | 0.080709633 | 3809 |
| KEGG_HUNTINGTONS_DISEASE | 159 | -1.222127308 | 0.039320078 | 0.080709633 | 6887 |
| KEGG_COLORECTAL_CANCER | 62 | -1.312946008 | 0.04102132 | 0.082821301 | 3427 |
| KEGG_HEDGEHOG_SIGNALING_PATHWAY | 56 | -1.368340289 | 0.042298374 | 0.083903989 | 3406 |
| KEGG_ERBB_SIGNALING_PATHWAY | 86 | -1.320775334 | 0.042920118 | 0.083903989 | 5194 |
| KEGG_CARDIAC_MUSCLE_CONTRACTION | 73 | -1.363481496 | 0.045876485 | 0.088151285 | 4986 |
| KEGG_INSULIN_SIGNALING_PATHWAY | 136 | -1.240415698 | 0.046524289 | 0.088151285 | 3355 |
| KEGG_BASAL_CELL_CARCINOMA | 55 | -1.359777569 | 0.049151904 | 0.091718864 | 5243 |

**Table 5b. RRM1 of GSEA analysis.**

| ID | setSize | enrichmentScore | NES | pvalue | qvalue | rank |
| --- | --- | --- | --- | --- | --- | --- |
| KEGG_PRIMARY_IMMUNODEFICIENCY | 35 | -0.65104027 | -2.158561822 | 5.98E-06 | 0.000894274 | 3100 |
| KEGG_LYSOSOME | 120 | -0.447268286 | -1.914846968 | 1.45E-05 | 0.001082557 | 3598 |
| KEGG_T_CELL_RECEPTOR_SIGNALING_PATHWAY | 107 | -0.432081898 | -1.834619705 | 6.95E-05 | 0.003461462 | 3100 |
| KEGG_OXIDATIVE_PHOSPHORYLATION | 101 | -0.430064517 | -1.806663125 | 0.000135651 | 0.004055241 | 4732 |
| KEGG_ALZHEIMERS_DISEASE | 145 | -0.391016303 | -1.733561803 | 0.000129362 | 0.004055241 | 4732 |
| KEGG_PARKINSONS_DISEASE | 99 | -0.429005877 | -1.794598166 | 0.000217893 | 0.005428208 | 5194 |
| KEGG_NITROGEN_METABOLISM | 23 | 0.657549668 | 1.984043384 | 0.000291924 | 0.005447192 | 4310 |
| KEGG_B_CELL_RECEPTOR_SIGNALING_PATHWAY | 75 | -0.464356569 | -1.855552349 | 0.000327982 | 0.005447192 | 3086 |
| KEGG_NATURAL_KILLER_CELL_MEDIATED_CYTOTOXICITY | 128 | -0.394895008 | -1.722250327 | 0.000297997 | 0.005447192 | 4584 |
| KEGG_FC_GAMMA_R_MEDIATED_PHAGOCYTOSIS | 94 | -0.402972188 | -1.664111701 | 0.000856502 | 0.012802446 | 4700 |
| KEGG_OTHER_GLYCAN_DEGRADATION | 16 | -0.695720114 | -1.933360957 | 0.00106006 | 0.01440465 | 3771 |
| KEGG_ACUTE_MYELOID_LEUKEMIA | 57 | -0.48033566 | -1.800557927 | 0.001180145 | 0.014700056 | 3850 |
| KEGG_HUNTINGTONS_DISEASE | 159 | -0.348098042 | -1.561566158 | 0.001504131 | 0.017294463 | 5286 |
| KEGG_FC_EPSILON_RI_SIGNALING_PATHWAY | 77 | -0.407948601 | -1.636752769 | 0.002812485 | 0.029066437 | 5461 |
| KEGG_HEMATOPOIETIC_CELL_LINEAGE | 85 | -0.398718225 | -1.622995672 | 0.002916878 | 0.029066437 | 3100 |
| KEGG_NON_SMALL_CELL_LUNG_CANCER | 54 | -0.443096055 | -1.646909449 | 0.004025598 | 0.037607561 | 3313 |
| KEGG_CELL_CYCLE | 123 | 0.363899192 | 1.534642365 | 0.005568942 | 0.048965314 | 3863 |
| KEGG_NOD_LIKE_RECEPTOR_SIGNALING_PATHWAY | 62 | 0.419623469 | 1.582638553 | 0.006137846 | 0.050969249 | 1933 |
| KEGG_GLYCOSAMINOGLYCAN_DEGRADATION | 20 | -0.57761024 | -1.695395683 | 0.007774245 | 0.058102255 | 4502 |
| KEGG_NOTCH_SIGNALING_PATHWAY | 47 | -0.444427035 | -1.588244427 | 0.007520392 | 0.058102255 | 4209 |
| KEGG_TYROSINE_METABOLISM | 40 | 0.47649928 | 1.644484434 | 0.00972429 | 0.063196761 | 2822 |
| KEGG_RIBOSOME | 86 | -0.378630682 | -1.546297201 | 0.009304574 | 0.063196761 | 6473 |
| KEGG_GAP_JUNCTION | 87 | 0.385296072 | 1.528221678 | 0.009446598 | 0.063196761 | 2016 |
| KEGG_HYPERTROPHIC_CARDIOMYOPATHY_HCM | 82 | 0.394707278 | 1.548428264 | 0.010583678 | 0.065915887 | 2427 |
| KEGG_CHEMOKINE_SIGNALING_PATHWAY | 186 | -0.299400677 | -1.377584115 | 0.011697126 | 0.069936504 | 1973 |
| KEGG_EPITHELIAL_CELL_SIGNALING_IN_HELICOBACTER_PYLORI_INFECTION | 67 | 0.404976094 | 1.5411552 | 0.014819731 | 0.085198454 | 1623 |
| KEGG_SYSTEMIC_LUPUS_ERYTHEMATOSUS | 53 | -0.414302447 | -1.530176543 | 0.015585029 | 0.086279693 | 2628 |
| KEGG_FATTY_ACID_METABOLISM | 42 | 0.460106476 | 1.61549385 | 0.01919126 | 0.102449585 | 2902 |
| KEGG_STARCH_AND_SUCROSE_METABOLISM | 38 | 0.460469368 | 1.579889703 | 0.021748493 | 0.10836091 | 4832 |
| KEGG_LONG_TERM_DEPRESSION | 66 | 0.384219265 | 1.461610027 | 0.021513111 | 0.10836091 | 2489 |
| KEGG_ARRHYTHMOGENIC_RIGHT_VENTRICULAR_CARDIOMYOPATHY_ARVC | 73 | 0.376290477 | 1.453267267 | 0.022856681 | 0.110208786 | 2427 |
| KEGG_BLADDER_CANCER | 41 | 0.431850314 | 1.507852277 | 0.025402351 | 0.116515343 | 2004 |
| KEGG_ASTHMA | 29 | -0.460860902 | -1.482270102 | 0.025723634 | 0.116515343 | 76 |
| KEGG_DRUG_METABOLISM_CYTOCHROME_P450 | 61 | 0.391803692 | 1.468852673 | 0.030978941 | 0.13619225 | 2713 |
| KEGG_ADHERENS_JUNCTION | 71 | 0.368868778 | 1.413569825 | 0.035823718 | 0.150500114 | 4254 |
| KEGG_COMPLEMENT_AND_COAGULATION_CASCADES | 68 | -0.359894703 | -1.40757798 | 0.036247211 | 0.150500114 | 1339 |
| KEGG_ANTIGEN_PROCESSING_AND_PRESENTATION | 81 | -0.353380046 | -1.43395793 | 0.043454598 | 0.164801795 | 3184 |
| KEGG_GLYCOLYSIS_GLUCONEOGENESIS | 61 | 0.380608243 | 1.426881487 | 0.042915493 | 0.164801795 | 4026 |
| KEGG_PPAR_SIGNALING_PATHWAY | 69 | 0.369077923 | 1.411429991 | 0.044849999 | 0.164801795 | 1947 |
| KEGG_CYTOKINE_CYTOKINE_RECEPTOR_INTERACTION | 256 | -0.261941521 | -1.250805237 | 0.045204436 | 0.164801795 | 3010 |
| KEGG_OLFACTORY_TRANSDUCTION | 372 | 0.252318447 | 1.210139417 | 0.04302926 | 0.164801795 | 4084 |

# Appendix 6

**Drug prediction**

**Table 6. Drug prediction.**

| search_term | gene | drug | interaction_types | sources |
| --- | --- | --- | --- | --- |
| CMPK1 | CMPK1 | GEMCITABINE | inhibitor | PharmGKB |
| CMPK1 | CMPK1 | LAMIVUDINE | unknown | PharmGKB |
| CMPK1 | CMPK1 | CISPLATIN | unknown | PharmGKB |
| NT5C2 | NT5C2 | DIDANOSINE | unknown | PharmGKB |
| NT5C2 | NT5C2 | GEMCITABINE | unknown | PharmGKB |
| NT5C2 | NT5C2 | MERCAPTOPURINE | unknown | CIViC|PharmGKB |
| NT5C2 | NT5C2 | THIOGUANINE | unknown | CIViC |
| NT5C2 | NT5C2 | CYTARABINE | unknown | PharmGKB |
| RRM1 | RRM1 | CLOFARABINE | inhibitor | TdgClinicalTrial|ChemblInteractions|TEND |
| RRM1 | RRM1 | GALLIUM NITRATE | inhibitor | ChemblInteractions |
| RRM1 | RRM1 | FLUDARABINE | inhibitor | TdgClinicalTrial|TEND |
| RRM1 | RRM1 | FLUDARABINE PHOSPHATE | inhibitor | ChemblInteractions |
| RRM1 | RRM1 | CISPLATIN | unknown | NCI|CIViC|PharmGKB |
| RRM1 | RRM1 | CLADRIBINE | inhibitor | PharmGKB |
| RRM1 | RRM1 | CYTARABINE | unknown | PharmGKB |
| RRM1 | RRM1 | HYDROXYUREA | inhibitor | TdgClinicalTrial|ChemblInteractions|TEND |
| RRM1 | RRM1 | DOCETAXEL | unknown | CIViC |
| RRM1 | RRM1 | PACLITAXEL | unknown | CIViC |
| RRM1 | RRM1 | GEMCITABINE | inhibitor | DTC|ClearityFoundationBiomarkers|TdgClinicalTrial|ClearityFoundationClinicalTrial|CIViC |
| RRM1 | RRM1 | TEZACITABINE | inhibitor | ChemblInteractions |
| RRM1 | RRM1 | GEMCITABINE HYDROCHLORIDE | inhibitor | ChemblInteractions |
| RRM1 | RRM1 | VINORELBINE | unknown | CIViC |

# Appendix 7

**MiRNA and LncRNA**

**Table 7a. Gene-miRNA.**

| Gene | miRNA | miRanda | miRDB | TargetScan | Sum |
| --- | --- | --- | --- | --- | --- |
| CMPK2 | hsa-miR-548h-5p | 1 | 1 | 1 | 3 |
| CMPK1 | hsa-let-7a-3p | 1 | 1 | 1 | 3 |
| CMPK2 | hsa-miR-374a-3p | 1 | 1 | 1 | 3 |
| CMPK2 | hsa-miR-202-5p | 1 | 1 | 1 | 3 |
| CMPK1 | hsa-miR-32-3p | 1 | 1 | 1 | 3 |
| CMPK1 | hsa-miR-506-3p | 1 | 1 | 1 | 3 |
| CMPK2 | hsa-miR-181c-5p | 1 | 1 | 1 | 3 |
| CMPK1 | hsa-miR-93-5p | 1 | 1 | 1 | 3 |
| CMPK1 | hsa-miR-3163 | 1 | 1 | 1 | 3 |
| CMPK2 | hsa-miR-4261 | 1 | 1 | 1 | 3 |
| CMPK2 | hsa-miR-559 | 1 | 1 | 1 | 3 |
| CMPK2 | hsa-miR-548i | 1 | 1 | 1 | 3 |
| CMPK2 | hsa-miR-4275 | 1 | 1 | 1 | 3 |
| CMPK1 | hsa-miR-106a-3p | 1 | 1 | 1 | 3 |
| CMPK2 | hsa-miR-548w | 1 | 1 | 1 | 3 |
| NT5C2 | hsa-miR-582-5p | 1 | 1 | 1 | 3 |
| NT5C2 | hsa-miR-4326 | 1 | 1 | 1 | 3 |
| CMPK1 | hsa-miR-548b-3p | 1 | 1 | 1 | 3 |
| CMPK2 | hsa-miR-4328 | 1 | 1 | 1 | 3 |
| CMPK1 | hsa-miR-548c-3p | 1 | 1 | 1 | 3 |
| NT5C2 | hsa-miR-505-5p | 1 | 1 | 1 | 3 |
| CMPK2 | hsa-miR-466 | 1 | 1 | 1 | 3 |
| CMPK1 | hsa-miR-495-3p | 1 | 1 | 1 | 3 |
| RRM1 | hsa-miR-551b-5p | 1 | 1 | 1 | 3 |
| CMPK1 | hsa-miR-512-3p | 1 | 1 | 1 | 3 |
| NT5C2 | hsa-miR-3170 | 1 | 1 | 1 | 3 |
| CMPK1 | hsa-miR-3180-5p | 1 | 1 | 1 | 3 |
| CMPK1 | hsa-miR-888-5p | 1 | 1 | 1 | 3 |
| NT5C2 | hsa-miR-519e-5p | 1 | 1 | 1 | 3 |
| NT5C2 | hsa-miR-96-3p | 1 | 1 | 1 | 3 |
| CMPK1 | hsa-miR-28-3p | 1 | 1 | 1 | 3 |
| CMPK1 | hsa-miR-4256 | 1 | 1 | 1 | 3 |
| CMPK1 | hsa-miR-106a-5p | 1 | 1 | 1 | 3 |
| CMPK1 | hsa-miR-15b-5p | 1 | 1 | 1 | 3 |
| CMPK2 | hsa-miR-4296 | 1 | 1 | 1 | 3 |
| CMPK2 | hsa-miR-548a-5p | 1 | 1 | 1 | 3 |
| NT5C2 | hsa-miR-1184 | 1 | 1 | 1 | 3 |
| CMPK1 | hsa-miR-542-3p | 1 | 1 | 1 | 3 |
| RRM1 | hsa-miR-514b-5p | 1 | 1 | 1 | 3 |
| CMPK2 | hsa-miR-2114-3p | 1 | 1 | 1 | 3 |
| CMPK2 | hsa-miR-181b-5p | 1 | 1 | 1 | 3 |
| CMPK1 | hsa-miR-130a-3p | 1 | 1 | 1 | 3 |
| CMPK1 | hsa-miR-195-5p | 1 | 1 | 1 | 3 |
| CMPK2 | hsa-miR-4287 | 1 | 1 | 1 | 3 |
| NT5C2 | hsa-miR-877-3p | 1 | 1 | 1 | 3 |
| RRM1 | hsa-miR-513c-5p | 1 | 1 | 1 | 3 |
| NT5C2 | hsa-miR-149-5p | 1 | 1 | 1 | 3 |
| CMPK1 | hsa-let-7f-1-3p | 1 | 1 | 1 | 3 |
| CMPK1 | hsa-miR-1323 | 1 | 1 | 1 | 3 |
| CMPK1 | hsa-miR-4272 | 1 | 1 | 1 | 3 |
| CMPK1 | hsa-miR-497-5p | 1 | 1 | 1 | 3 |
| NT5C2 | hsa-miR-548t-5p | 1 | 1 | 1 | 3 |
| RRM1 | hsa-miR-205-3p | 1 | 1 | 1 | 3 |
| NT5C2 | hsa-miR-181a-2-3p | 1 | 1 | 1 | 3 |
| CMPK1 | hsa-miR-143-3p | 1 | 1 | 1 | 3 |
| CMPK1 | hsa-miR-1236-3p | 1 | 1 | 1 | 3 |
| NT5C2 | hsa-miR-3123 | 1 | 1 | 1 | 3 |
| NT5C2 | hsa-miR-381-3p | 1 | 1 | 1 | 3 |
| CMPK1 | hsa-miR-15a-5p | 1 | 1 | 1 | 3 |
| CMPK2 | hsa-miR-33a-3p | 1 | 1 | 1 | 3 |
| NT5C2 | hsa-miR-3120-3p | 1 | 1 | 1 | 3 |
| CMPK1 | hsa-miR-503-5p | 1 | 1 | 1 | 3 |
| CMPK1 | hsa-miR-1178-3p | 1 | 1 | 1 | 3 |
| CMPK1 | hsa-miR-16-5p | 1 | 1 | 1 | 3 |
| CMPK1 | hsa-miR-573 | 1 | 1 | 1 | 3 |
| CMPK2 | hsa-miR-944 | 1 | 1 | 1 | 3 |
| CMPK2 | hsa-miR-140-3p | 1 | 1 | 1 | 3 |
| CMPK1 | hsa-miR-20b-5p | 1 | 1 | 1 | 3 |
| CMPK2 | hsa-miR-760 | 1 | 1 | 1 | 3 |
| NT5C2 | hsa-miR-300 | 1 | 1 | 1 | 3 |
| NT5C2 | hsa-miR-20b-3p | 1 | 1 | 1 | 3 |
| CMPK2 | hsa-miR-3165 | 1 | 1 | 1 | 3 |
| CMPK2 | hsa-miR-3122 | 1 | 1 | 1 | 3 |
| NT5C2 | hsa-miR-548m | 1 | 1 | 1 | 3 |
| NT5C2 | hsa-miR-885-5p | 1 | 1 | 1 | 3 |
| CMPK1 | hsa-miR-4282 | 1 | 1 | 1 | 3 |
| RRM1 | hsa-miR-33a-3p | 1 | 1 | 1 | 3 |
| CMPK1 | hsa-miR-29b-3p | 1 | 1 | 1 | 3 |
| RRM1 | hsa-miR-4328 | 1 | 1 | 1 | 3 |
| CMPK2 | hsa-miR-32-3p | 1 | 1 | 1 | 3 |
| NT5C2 | hsa-miR-1261 | 1 | 1 | 1 | 3 |
| RRM1 | hsa-miR-101-3p | 1 | 1 | 1 | 3 |
| CMPK2 | hsa-miR-548c-5p | 1 | 1 | 1 | 3 |
| CMPK1 | hsa-miR-330-3p | 1 | 1 | 1 | 3 |
| CMPK1 | hsa-miR-767-5p | 1 | 1 | 1 | 3 |
| RRM1 | hsa-miR-338-3p | 1 | 1 | 1 | 3 |
| CMPK1 | hsa-miR-301b-3p | 1 | 1 | 1 | 3 |
| CMPK2 | hsa-miR-4322 | 1 | 1 | 1 | 3 |
| CMPK1 | hsa-miR-133b | 1 | 1 | 1 | 3 |
| NT5C2 | hsa-miR-4277 | 1 | 1 | 1 | 3 |
| CMPK1 | hsa-miR-29a-3p | 1 | 1 | 1 | 3 |
| CMPK1 | hsa-miR-3120-3p | 1 | 1 | 1 | 3 |
| CMPK2 | hsa-miR-1205 | 1 | 1 | 1 | 3 |
| NT5C2 | hsa-miR-409-3p | 1 | 1 | 1 | 3 |
| NT5C2 | hsa-miR-1236-3p | 1 | 1 | 1 | 3 |
| CMPK2 | hsa-miR-548d-5p | 1 | 1 | 1 | 3 |
| CMPK2 | hsa-miR-3202 | 1 | 1 | 1 | 3 |
| CMPK1 | hsa-miR-302d-5p | 1 | 1 | 1 | 3 |
| NT5C2 | hsa-miR-515-5p | 1 | 1 | 1 | 3 |
| NT5C2 | hsa-miR-136-5p | 1 | 1 | 1 | 3 |
| CMPK2 | hsa-miR-548b-5p | 1 | 1 | 1 | 3 |
| CMPK1 | hsa-miR-302b-5p | 1 | 1 | 1 | 3 |
| CMPK1 | hsa-miR-106b-5p | 1 | 1 | 1 | 3 |
| CMPK2 | hsa-miR-548c-3p | 1 | 1 | 1 | 3 |
| CMPK1 | hsa-miR-195-3p | 1 | 1 | 1 | 3 |
| CMPK1 | hsa-miR-16-2-3p | 1 | 1 | 1 | 3 |
| NT5C2 | hsa-miR-3137 | 1 | 1 | 1 | 3 |
| CMPK2 | hsa-miR-3143 | 1 | 1 | 1 | 3 |
| CMPK1 | hsa-miR-3133 | 1 | 1 | 1 | 3 |
| NT5C2 | hsa-miR-578 | 1 | 1 | 1 | 3 |
| NT5C2 | hsa-miR-1205 | 1 | 1 | 1 | 3 |
| CMPK1 | hsa-miR-130b-3p | 1 | 1 | 1 | 3 |
| CMPK2 | hsa-miR-181a-5p | 1 | 1 | 1 | 3 |
| NT5C2 | hsa-miR-4329 | 1 | 1 | 1 | 3 |
| CMPK1 | hsa-miR-424-5p | 1 | 1 | 1 | 3 |
| CMPK2 | hsa-miR-522-3p | 1 | 1 | 1 | 3 |
| CMPK1 | hsa-miR-646 | 1 | 1 | 1 | 3 |
| CMPK2 | hsa-miR-3199 | 1 | 1 | 1 | 3 |
| CMPK1 | hsa-miR-29c-3p | 1 | 1 | 1 | 3 |
| CMPK1 | hsa-let-7b-3p | 1 | 1 | 1 | 3 |
| CMPK1 | hsa-miR-758-3p | 1 | 1 | 1 | 3 |
| CMPK2 | hsa-miR-4265 | 1 | 1 | 1 | 3 |
| NT5C2 | hsa-miR-495-3p | 1 | 1 | 1 | 3 |

**Table 7b. Gene-lncRNA.**

| miRNA | lncRNA |
| --- | --- |
| hsa-miR-767-5p | RP11-326C3.10 |
| hsa-miR-515-5p | TTLL10-AS1 |
| hsa-miR-136-5p | CTD-2534I21.9 |
| hsa-miR-149-5p | LINC01043 |
| hsa-miR-767-5p | RP11-326C3.14 |
| hsa-miR-515-5p | AC079779.7 |
| hsa-miR-130a-3p | CITF22-1A6.3 |
| hsa-let-7a-3p | RP3-323A16.1 |
| hsa-miR-338-3p | RP11-229P13.22 |
| hsa-miR-1184 | TMEM191C |
| hsa-miR-1178-3p | AC011284.3 |
| hsa-let-7a-3p | DPP10-AS2 |
| hsa-miR-101-3p | AC091153.4 |
| hsa-miR-338-3p | RP4-539M6.22 |
| hsa-miR-515-5p | RP13-507P19.2 |
| hsa-miR-130a-3p | LA16c-306A4.2 |
| hsa-miR-136-5p | RP11-526P6.1 |
| hsa-miR-1184 | LINC00689 |
| hsa-miR-140-3p | RP11-326C3.10 |
| hsa-miR-512-3p | RP11-717I24.1 |
| hsa-miR-140-3p | RP11-210M15.1 |
| hsa-miR-1236-3p | RP11-717I24.1 |
| hsa-miR-877-3p | LINC00689 |
| hsa-miR-149-5p | RP11-394A14.2 |
| hsa-miR-338-3p | CTD-2292P10.2 |
| hsa-miR-338-3p | LINC01165 |
| hsa-let-7a-3p | FAM230B |
| hsa-miR-181a-2-3p | RP3-470B24.5 |
| hsa-miR-1184 | RP3-470B24.5 |
| hsa-miR-505-5p | CTB-51J22.1 |
| hsa-miR-181a-2-3p | FLJ16779 |
| hsa-miR-181a-5p | LL22NC03-27C5.1 |
| hsa-miR-515-5p | RP13-580B18.4 |
| hsa-miR-542-3p | RP4-671O14.7 |
| hsa-miR-1184 | HCG22 |
| hsa-miR-338-3p | RP11-780K2.1 |
| hsa-miR-512-3p | RP1-182D15.2 |
| hsa-miR-338-3p | PCBP3-OT1 |
| hsa-miR-542-3p | AC079586.1 |
| hsa-miR-140-3p | CITF22-1A6.3 |
| hsa-miR-542-3p | RP11-157B13.7 |
| hsa-miR-1236-3p | LINC00689 |
| hsa-miR-877-3p | LINC00940 |
| hsa-miR-1236-3p | LINC00940 |
| hsa-miR-515-5p | SPACA6P |
| hsa-miR-515-5p | AC093642.4 |
| hsa-miR-130a-3p | SNHG14 |
| hsa-miR-106a-5p | LINC01106 |
| hsa-miR-542-3p | LINC00917 |
| hsa-miR-1184 | RP11-1260E13.4 |
| hsa-miR-338-3p | MCF2L-AS1 |
| hsa-miR-106a-5p | RP11-369C8.1 |
| hsa-miR-1184 | AC092535.3 |
| hsa-miR-181a-2-3p | RP11-368I7.4 |
| hsa-miR-140-3p | LA16c-306A4.2 |
| hsa-miR-338-3p | LINC01423 |
| hsa-miR-515-5p | FAM74A1 |
| hsa-miR-505-5p | AC016682.1 |
| hsa-miR-140-3p | LINC01128 |
| hsa-miR-1184 | TMEM191A |
| hsa-miR-181a-2-3p | RP11-830F9.5 |
| hsa-miR-140-3p | LINC00854 |
| hsa-miR-140-3p | RP11-326C3.14 |
| hsa-miR-181a-2-3p | FAM95B1 |
| hsa-miR-758-3p | AC079586.1 |
| hsa-miR-338-3p | AC015849.16 |
| hsa-miR-515-5p | LINC01002 |
| hsa-miR-542-3p | LINC01224 |
| hsa-miR-338-3p | SNHG14 |
| hsa-miR-136-5p | SPACA6P |
| hsa-miR-1184 | CTD-3193O13.11 |
| hsa-miR-760 | AC006019.3 |
| hsa-miR-1236-3p | RP11-798K23.1 |
| hsa-miR-29a-3p | RP11-223P11.3 |
| hsa-miR-767-5p | RP11-223P11.3 |
| hsa-miR-758-3p | LINC01224 |
| hsa-miR-1236-3p | RP11-91K11.2 |
| hsa-miR-760 | RP11-394A14.2 |
| hsa-miR-1184 | RP1-29C18.10 |
| hsa-miR-1236-3p | RP11-23J9.4 |
| hsa-miR-515-5p | CTD-2197I11.1 |
| hsa-miR-15a-5p | RP11-483P21.6 |
| hsa-miR-767-5p | MCF2L-AS1 |
| hsa-miR-515-5p | RP11-1217F2.15 |
| hsa-miR-1184 | CTD-2311B13.1 |
| hsa-miR-888-5p | AC079799.2 |
| hsa-miR-130a-3p | RP4-539M6.22 |
| hsa-miR-512-3p | RP5-1154L15.1 |
| hsa-miR-149-5p | CTD-2008P7.3 |
| hsa-miR-338-3p | CTD-3138B18.5 |
| hsa-miR-32-3p | TCF4-AS2 |
| hsa-miR-515-5p | CTD-3099C6.5 |
| hsa-miR-1184 | AC139099.4 |
| hsa-miR-338-3p | GRM7-AS1 |
| hsa-miR-515-5p | SNHG14 |
| hsa-miR-877-3p | RP1-253P7.1 |
| hsa-miR-515-5p | AC084219.4 |
| hsa-miR-1184 | COL18A1-AS1 |
| hsa-miR-515-5p | AC015849.16 |
| hsa-miR-1184 | RP3-402G11.28 |
| hsa-miR-106a-3p | LLPH-AS1 |
| hsa-miR-944 | RP5-1077H22.2 |
| hsa-miR-374a-3p | PKD1P6 |
| hsa-let-7a-3p | LPP-AS2 |
| hsa-miR-515-5p | FAM74A7 |
| hsa-miR-28-3p | LINC00662 |
| hsa-miR-758-3p | RP11-157B13.7 |
| hsa-miR-767-5p | RP4-539M6.22 |
| hsa-miR-758-3p | RP11-15H20.6 |
| hsa-miR-181a-5p | ZNF833P |
| hsa-miR-140-3p | AC064853.2 |
| hsa-miR-1205 | RP3-470B24.5 |
| hsa-miR-15a-5p | RP11-34P13.7 |
| hsa-miR-143-3p | LL22NC03-2H8.5 |
